# Construction of a Prognostic Model for Cervical Cancer Related to lncRNA Based on Differential Co-expression Network and Functional Study of Key Gene EGFR-AS1

**DOI:** 10.7150/jca.108429

**Published:** 2025-03-31

**Authors:** Kailong Du, Qian Chen, Hui Fan, Yunlong Lei, Jian Zhang

**Affiliations:** 1 Department of Biochemistry and Molecular Biology, College of Basic Medical Sciences, Chongqing Medical University, Chongqing 400016, China.; 2 Molecular Medicine and Cancer Research Center, Chongqing Medical University, Chongqing 400016, China.; 3 Frontiers Medical Center, Tianfu Jincheng Laboratory, Chengdu 610212, China.

**Keywords:** cervical cancer, lncRNA, co-expression network, EGFR-AS1, FAM83B

## Abstract

Cervical cancer is a common gynecological malignancy, and the average age of onset is decreasing gradually. Therefore, an effective predictive model is urgently needed to improve the personalized treatment of cervical cancer patients. Long non-coding RNAs (lncRNAs) play crucial roles in the occurrence, development, and prognosis of malignant tumors. In this study, we used cervical cancer multi-omics data and single-cell sequencing data for analysis, and established a 33-lncRNA-CESC model by using the random forest algorithm in ensemble learning and mRNA and lncRNA co-expression network technology. The results demonstrated that the model exhibited strong discriminative ability, accuracy, and clinical utility. Furthermore, we investigated the relationship between the model and immune cell infiltration. Enrichment analysis revealed associations between the model and cellular proliferation as well as epidermal growth factor receptor (EGFR) signaling pathways. Subsequently, attention was directed toward the gene EGFR-AS1 in the model, which was identified within the co-expression network and exhibited a significant association with patient prognosis. Additionally, EGFR-AS1 was found to be specifically associated with FAM83B. Analysis of single-cell data confirmed that FAM83B plays a role in the late stage of cervical cancer development mainly through the EGFR signaling pathway. Functional experiments showed that knockdown of either EGFR-AS1 or FAM83B inhibited cervical cancer cell proliferation and migration capabilities, and the phosphorylated ERK and AKT levels. In addition, there was a mutual regulatory effect between EGFR-AS1 and FAM83B expression. In conclusion, this study identifies that EGFR-AS1 served as a key factor in our 33-lncRNA-CESC model and potentially interacted with FAM83B to regulate the EGFR pathway which significantly impacting cervical cancer development.

## Introduction

Cervical cancer is one of the most common cancers among women, ranking fourth in terms of incidence and mortality among malignant tumors in females. In 2020, there were an estimated 604,127 cases of cervical cancer and 341,831 deaths globally^1^, with China accounting for 18% and 17% of the incidence and mortality rates, respectively^2^. Standard treatments for cervical cancer include radiotherapy, chemotherapy, and surgery. Although some progress has been made in recent years, the long-term prognosis for cervical cancer remains poor due to drug resistance and recurrence^3^. Therefore, exploring new effective biomarkers and treatments is crucial for improving the prognosis of cervical squamous cell carcinoma (CESC) patients.

Long non-coding RNAs (lncRNAs) are RNA molecules longer than 200 nucleotides that do not encode proteins but possess specific functions such as mRNA splicing, transcriptional regulation, and post-transcriptional regulation^4^. Numerous studies have demonstrated the indispensable role of lncRNA in the initiation and progression of tumors, with the potential to alter tumor characteristics and induce variations in the tumor microenvironment^5^. Their excellent molecular stability makes them as potential novel prognostic biomarkers for cancer patients 6,7. Previous studies have investigated the role of lncRNAs in cervical cancer. Zhong et al. identified 8 lncRNA markers for cervical cancer by integrating DNA methylation, copy number variation, and transcriptome data 8. Zhang et al. found that the LINC00240/microRNA-124-3p/STAT3/MICA axis promotes cytotoxic activity of natural killer T cells in cervical cancer^9^. These studies have demonstrated the key role of lncRNAs in the development of cervical cancer, and have also provided some insights for clinical treatment using routine data and methods. However, there is currently no report on studying cervical cancer using co-expression networks and single-cell data combined with random forest model. Therefore, further elucidating the relationship between lncRNAs and CESC is of crucial significance in exploring novel therapeutic models for CESC and improving patient prognosis.

Epidermal Growth Factor (EGF) is an important growth factor that plays a crucial role in cell proliferation^10^. In cancer, EGF and EGFR are often found to be abnormally overexpressed, and EGFR is currently overexpressed in 54-74% of cervical cancer^10^,^11^. Transcription of EGFR-AS1 occurs on the antisense strand of EGFR, exhibiting partial sequence complementarity with EGFR. Studies have shown knockdown of EGFR-AS1 reduces EGFR expression by decreasing EGFR mRNA stability^12^. Overexpression of EGFR-AS1 in non-small cell lung cancer is associated with poor prognosis and promotes chemotherapy resistance^13^. EGFR-AS1 also mediates EGFR addiction and modulates treatment response in squamous cell carcinoma^14^. EGFR-AS1 promotes renal cancer cell growth and metastasis by influencing the stability of EGFR mRNA mediated by HuR^15^. However, the role and mechanism of EGFR-AS1 in CESC have not been reported, and its regulatory relationship with other genes remains unknown.

In this study, we first systematically analyzed and identified the 33-lncRNA-CESC model at the transcriptome level, and found that the 33-lncRNA-CESC model could accurately predict the prognosis of CESC patients. We further demonstrated that the key lncRNA EGFR-AS1 could interact with FAM83B and play an important role in cervical cancer by regulating the EGFR pathway.

## Materials and Methods

### Data acquisition and preprocessing

Human lncRNA GTF annotation file was retrieved from GENCODE website to identify lncRNA^16^. The lncRNA expression profile file obtained according to the annotation file is shown in supplementary Table 1. CESC transcriptome data and clinical data were downloaded from The Cancer Genome Atlas (TCGA) data portal 17, and the clinic characteristics of the 309 patients are shown in supplementary Table S2. Transcriptome RNA-seq and clinical data from 306 CESC patients were collected from the TCGA database. Mutation data for 289 CESC samples were downloaded from the TCGA database in MAF format. To facilitate the follow-up analysis, the gene expression matrix was filtered based on the following criteria: the genes that show null expression values in more than 30 samples are removed, and then the genes without corresponding annotation information were also removed. The single-cell sequencing data GSE197461 was downloaded from the GEO database, comprising three cervical squamous cell carcinoma samples, totaling 16,312 single-cells. The entire process was analyzed by R (version 4.2.1).

### Identification of CESC-related lncRNA

First, differential analysis on the TCGA-CESC transcriptome was conducted using the 'EDSeq2' package, with the criteria for differential gene selection set at |log2FC| > 1 and adjust P value < 0.05. Then, correlation analysis was performed between the differentially expressed genes in the transcriptome and all annotated lncRNAs, with the criteria set at a Pearson correlation coefficient > 0.5 and P value < 0.05.

### Mutation analysis and network visualization

The TCGA-CESC mutation data (including CNV) was analyzed using the GISTIC2 software. To demonstrate the mutually regulated connection between lncRNA and corresponding target mRNAs, the co-expression network of differentially expressed genes and lncRNAs from the transcriptome was visualized using the Cytoscape software. The protein-protein interaction (PPI) was analyzed with STRING by setting the minimum interaction sources as 0.400.

### Functional enrichment analysis

In order to understand the biological processes potentially involved in the co-expression network, the DAVID v6.8 (The Database for Annotation, Visualization and Integrated Discovery) were utilized to conduct pathway analysis. To clarify the differences in enriched pathways between the low-risk and high-risk subgroups, Gene Set Enrichment Analysis (GSEA) was performed using Kyoto Encyclopedia of Genes and Genomes (KEGG) pathway gene sets and Reactome pathway gene sets.

### Construction and assessment of prognostic risk score model for CESC

Univariate Cox proportional regression analysis was applied to determine the prognosis-related lncRNA (p < 0.05) in TCGA-CESC. The 'randomForestSRC' R package was used to establish the lncRNA random forest model related to prognosis, and was verified by bootstrap. Patients were divided into high risk and low risk groups using the median risk score obtained by the constructed model in the training set as the cutoff point, and the formula was established as follows:

risk score = ExplncRNA1× βlncRNA1+ ExplncRNA2×βlncRNA2+ ... + ExplncRNAn× βlncRNA, (1) ExpmRNA represents the expression level of each lncRNA, and βmRNA denotes the importance coefficient of the lncRNA in the Random forest model. Subsequently, time-dependent Receiver Operating Characteristic (ROC) curves and Kaplan-Meier (K-M) curves were plotted to evaluate survival prediction, and the area under the ROC curve (AUC) was calculated to assess the predictive accuracy and specificity of the features.

### Nomogram establishment and the correlation between the prognostic signature and clinical characteristics

To validate the independence of the lncRNA model, we conducted multivariable Cox regression analysis to assess the relationship between the lncRNA model and overall survival rate. Subsequently, we investigated whether the features could be considered as independent risk factors from other clinical-pathological factors (such as grade, stage, TNM staging, etc.). In order to predict the overall survival rates of lung adenocarcinoma patients for 1 to 3 years, a nomogram considering independent prognostic factors was plotted using the 'rms' package. Additionally, calibration curves were drawn to compare the consistency of the predicted 1-, 2-, and 3-year survival probabilities based on the line chart with the actual observations. Finally, a series of net benefit probabilities for risk threshold was calculated using the 'rmda' package, and decision curve analysis (DCA) curves were plotted to assess the clinical effectiveness of the nomogram.

### Immune cell infiltration analysis

The composition of immune cells and stromal cells in the tumor microenvironment (TME) was calculated for each CESC sample. The ESTIMATE algorithm was used to validate differences in TME features between different risk groups. The 'CIBERSORT' package was employed to extract the relative proportions of 22 human infiltrating immune cells, which can reveal the correlation between risk features and immune cell characteristics. Additionally, single-sample gene set enrichment analysis (ssGSEA) scores were used to evaluate the enrichment levels of 29 immune-related functions between the two groups.

### Single-cell data analysis

Sample integration was performed using the 'harmony' package (v0.1.1), followed by data transformation into Seurat objects using the 'Seurat' package (v4.3.0) for quality control. Quality control criteria for the samples were as follows: expression of all genes in at least three cells, each cell expressing at least 50 genes, and a proportion of mitochondrial genes less than 15%. Subsequently, the findclusters function in Seurat was utilized to identify major cell clusters. Clustering results were visualized using the UMAP (Uniform Manifold Approximation and Projection) method. Cell type annotation was then conducted using the 'SingleR' package (v1.10.0). Copy number variations within cell clusters were analyzed using the 'inferCNV' package (v1.12.0), using the following parameters: "Denoising", default Hidden Markov Model (HMM) setting, "Cutoff" is 0.1. To reduce the possibility of false positives, CNV calls to the default Bayesian latent hybrid model are implemented to identify the posterior probability of changes in each cell. The default threshold 0.5 is used to filter low probability CNVS. The trajectory of sub-clustered epithelial cells was delineated using monocle2 (version 2.26.0). Subsequently, significant clusters underwent Gene Set Variation Analysis (GSVA).

### Cell culture, siRNAs, and transfection

Human CESC cell lines, HeLa, was obtained from Chinese Academy of Sciences Shanghai cell bank (Shanghai, China). Cells were routinely maintained in DMEM medium (Hyclone) supplemented with 10% of fetal bovine serum (Hyclone), penicillin (10^7^ U/L) and streptomycin (10 mg/L) in a humidified incubator containing 5% CO_2_ at 37 °C. Cell lines were routinely tested for short tandem repeat authentication and mycoplasma contamination 18. The short interfering RNAs (siRNA) were chemically synthesized by TsingkeBiotechnology (Beijing, China), and were transiently transfected into cells using Lipofectamine 3000 reagent (Invitrogen). The sequences of the siRNAs were provided in supplementary Table S3.

### qRT-PCR and immunoblotting

Total RNA was extracted from cells by using Trizol reagent (Invitrogen) and qRT-PCR was performed by using SYBR PremixEx TaqTM (TaKaRa) as previously described^18^. The primer sequences are provided in supplementary Table S3.

For immunoblotting, cells were lysed with RIPA buffer (Beyotime) supplemented with protease inhibitor cocktail (Sigma) and then protein lysates were centrifuged and boiled with loading buffer. All lysates were quantified by the BCA Protein Assay (Thermo Fisher Scientific) and analyzed by SDS-PAGE. Antibodies were shown in supplementary Table S4.

### Cell proliferation, migration and viability assays

Cell viability was determined by CCK8 method. Simply, the cells are inoculated on a 96-well plate at a density of 2,000 cells. After treatment, CCK8 (Bimake, B34302) reagent was added and incubated for 2h. Then the absorbance was measured at 450 nm.

EdU labelling assays were conducted by using Cell-Light EdU Apollo488 In vitro Kit (C10310-3, RiboBio, China) following the manufacturer's instructions. Briefly, the exponentially growing cells were seeded on 96-well plates and then sequentially labelled with 10 μM EdU for 2h, fixed, stained with Apollo488 and Hoechst 33342 (DNA dye for nuclei staining), and finally observed under fluorescence microscope (Leica, Germany).

For Transwell cell migration assays, HeLa cells were seeded in the upper chamber of a Transwell device (8 µm, Merck Millipore, Darmstadt, Germany) at a density of 2 × 10^4^ (for migration) cells per well in serum-free, and 700 μL of medium containing 5% FBS was added to the bottom chamber. Thirty-six hours later, the cells were fixed with methyl alcohol for 20 min, and stained with 0.5% crystal violet for 10 min. Then the cells on the upper surface of the filter were removed using a cotton swab. Five fields were imaged per Transwell insert, and the number of cells was counted using the particle counting module in ImageJ V1.54f.

### Molecular interaction analysis

To explore the potential interactions of lncRNA, the likelihood of binding between lncRNA and target protein was predicted using the catRAPID algorithm 19. Subsequently, the software Risearch2^20^ was used to analyze the potential interactions between lncRNAs and target genes.

### Data analysis and statistics

Data were expressed as means ± sd. All experiments were performed at least three times. Statistical analysis was performed with GraphPad Prism 8.0 software. Statistical differences between groups were determined using two-tailed Student's t-test. Significance was designated as follows: *, P < 0.05, **, P < 0.01, ***, P < 0.001.

## Results

### The genes of CESC differentially co-expressed network influence the development of cervical cancer through hat acetylated histone and other pathways

**Figure 1:**
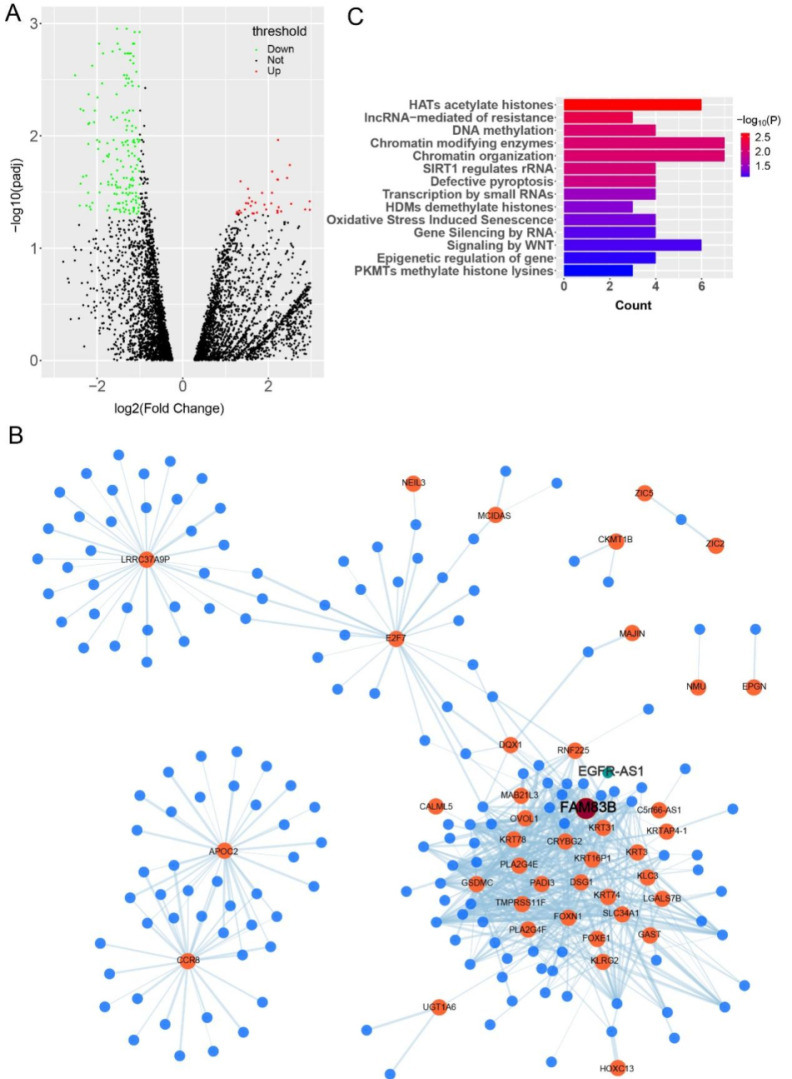
**The co-expression network reveals the possible regulatory relationships and pathways involved in cervical cancer.** (A) The 'DEseq2' package was used to analyze the difference of transcriptome data of 306 patients with cervical cancer in TCGA database and generate volcano map. (B) The DAVID online analysis website was used to conduct pathway enrichment analysis of differential genes. The figure shows the main pathways involved in the co-expression network. (C) The co-expression network of differentially expressed genes and lncRNAs from the transcriptome was visualized using the Cytoscape software. The orange dots represent mRNAs, the blue dots represent lncRNAs, and the connected edges indicate that there is a correlation between them.

In order to reveal the genetic differences between cervical cancer and normal tissues, 306 cervical cancer transcriptome samples downloaded from TCGA database were used for differential analysis (the research process is shown in supplementary Figure 1). A total of 268 differentially expressed genes were found, including 78 up-regulated genes and 190 down-regulated genes (Figure 1A, supplementary Table S5). PPI network analysis results showed that the differential genes in histone H1 family, SCN family, synaptic binding protein family, etc. may play a role in the progression of cervical cancer (supplementary Figure 2). Studies have shown that histone H1 may act as a tumor suppressor^21^. The SCN family plays a regulatory role in hormone and growth factor-related cancer development^22^, which is consistent with our findings. Then, using these 268 differentially expressed genes and 4297 lncRNAs annotated from the GENCODE database for Pearson correlation analysis, we constructed a co-expression network of mRNA and lncRNA (Figure 1B, Supplementary table S6). In order to study the pathways involved in the network, we performed pathway enrichment analysis of the genes in the network. The results showed that genes in the co-expression network were involved in hat acetylated histone, lncRNA-mediated resistance, DNA methylation, chromatin modifying enzymes and other pathways (Figure 1C).

### 33-lncRNA-CESC model can significantly predict the prognosis of CESC patients

**Figure 2:**
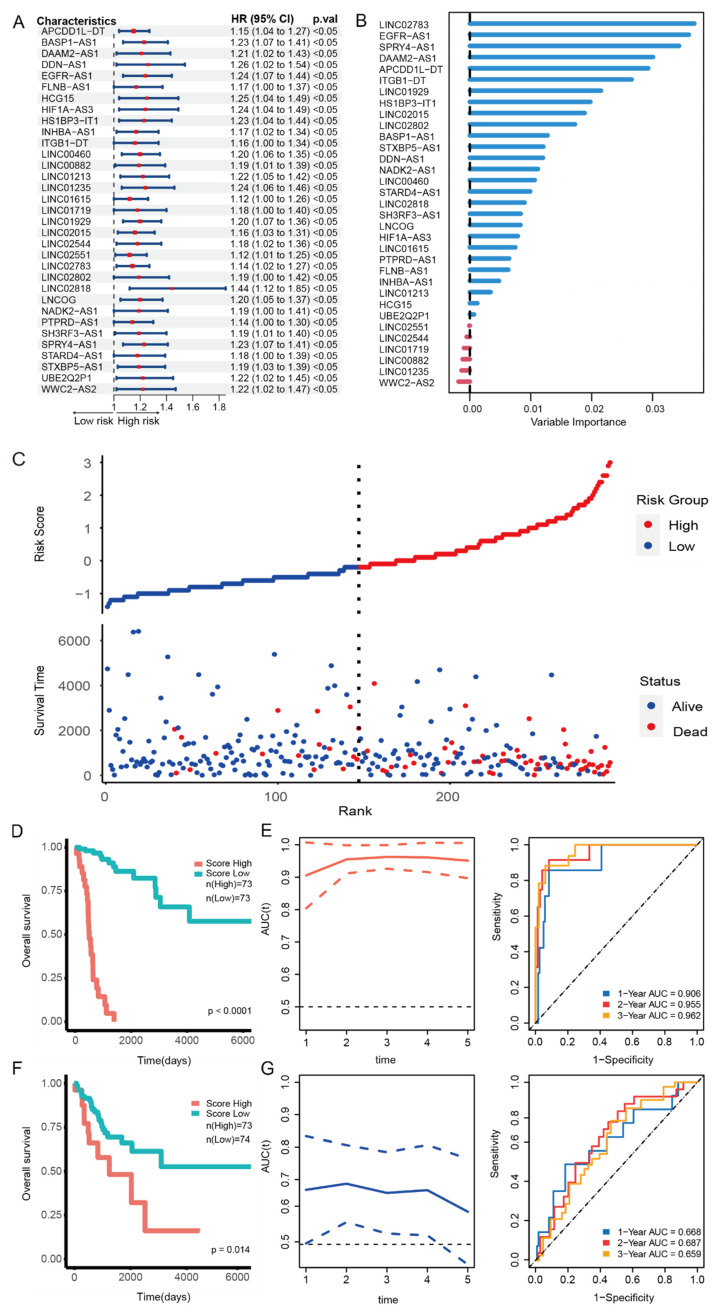
**33-lncRNA-CESC model is significantly associated with the prognosis of cervical cancer patients.** (A) Univariate Cox regression analysis was used for 1308 lncRNAs annotated in GENCODE database, and forest-plot were drawn for 33 lncRNAs with 'forestploter' package after screening (p < 0.05). (B) The 'randomForestSRC' package was used to construct a random forest model for the 33 lncRNAs selected in the previous step. The importance of each variable was ranked as shown in the figure. (C) The 'randomForestSRC' package was used to generate the scatterplot of risk score and survival status in 293 CESC patients. (D.F) The 'survival' package in R software was used to draw Kaplan-Meier curves for groups with high and low risk scores, and log-rank was used to test whether the prediction ability of the evaluation model was significant (P<0.05). (E.G) The 'timeROC' package was used to plot the ROC curve of 1, 2, and 3-year overall survival for CESC patients with survival data and model risk scores. (E) 50%TCGA-CESC samples were selected by random sampling as the training set for ROC analysis. (G) The remaining 50% samples were used as the test set for ROC analysis.

In order to predict whether a single lncRNA might be a prognostic factor for CESC, we used univariate Cox regression analysis to screen out 33 lncRNAs that were significantly correlated with overall survival (OS) of CESC patients from the 1308 lncRNAs annotated in the GENCODE database (Figure 2A). These lncRNAs can act as independent risk factors for the prognosis of CESC patients. Subsequently, in order to quantify the weight of 33 lncRNAs in the model to predict cervical cancer prognosis, we randomly selected 50% of the data as a training set to build a random forest model.

The importance of each lncRNA in the model is shown in Figure 2B, and the specific coefficients are listed in supplementary table S7. Interestingly, the lncRNAs with the high weights in the model have all been reported to be related to the occurrence and development of tumors. For example, LINC02738 may be involved in the occurrence and development of renal cancer through the miR-20b/Sox4 axis^23^. SPRY4-AS1, a novel enhancer RNA, may be a new target for hepatocellular carcinoma^24^. EGFR-AS1, which ranked second in weight, was reported to be involved in the process of multiple cancers^12^-^15^. These results indicate that the model can successfully predict the prognosis of patients with cervical cancer.

In order to test the ability of the 33-lncRNA-CESC model to predict CESC patient survival, patients were divided into high-risk group and low-risk group according to the median risk score calculated by the weights of each lncRNA in the 33-lncRNA-CESC model, and scatter plots were drawn. Results as shown in Figure 2C, the number of deaths increased significantly with the increase of risk score, indicating that the model risk score can significantly predict the survival of CESC patients. We then use the 'Survival' package in R software to draw Kaplan-Meier curves to evaluate the model's predictive ability. Patients in the high-risk group exhibited significantly poorer survival outcomes compared to those in the low-risk group (Figure 2D). The ROC curve demonstrated the predictive ability of the risk score for prognosis, with area under the curve (AUC) values of 0.906, 0.955, and 0.962 for 1-year, 2-year, and 3-year survival, respectively (Figure 2E), which indicate that the 33-lncRNA-CESC model has a very significant ability to predict the prognosis of the training set. Subsequently, the 33-lncRNA-CESC model was tested using the remaining 50% of the data as the testing set, and the analysis was repeated. Consistently, patients with low-risk scores had better prognosis (Figure 2F). The risk score demonstrated good prognostic predictive capability, with AUC values of 0.668, 0.687, and 0.659 for 1-year, 2-year, and 3-year survival, respectively (Figure 2G). These results show that the 33-lncRNA-CESC model has good generalization ability.

### Nomogram based on the 33-lncRNA-CESC model has clinical applicability

**Figure 3:**
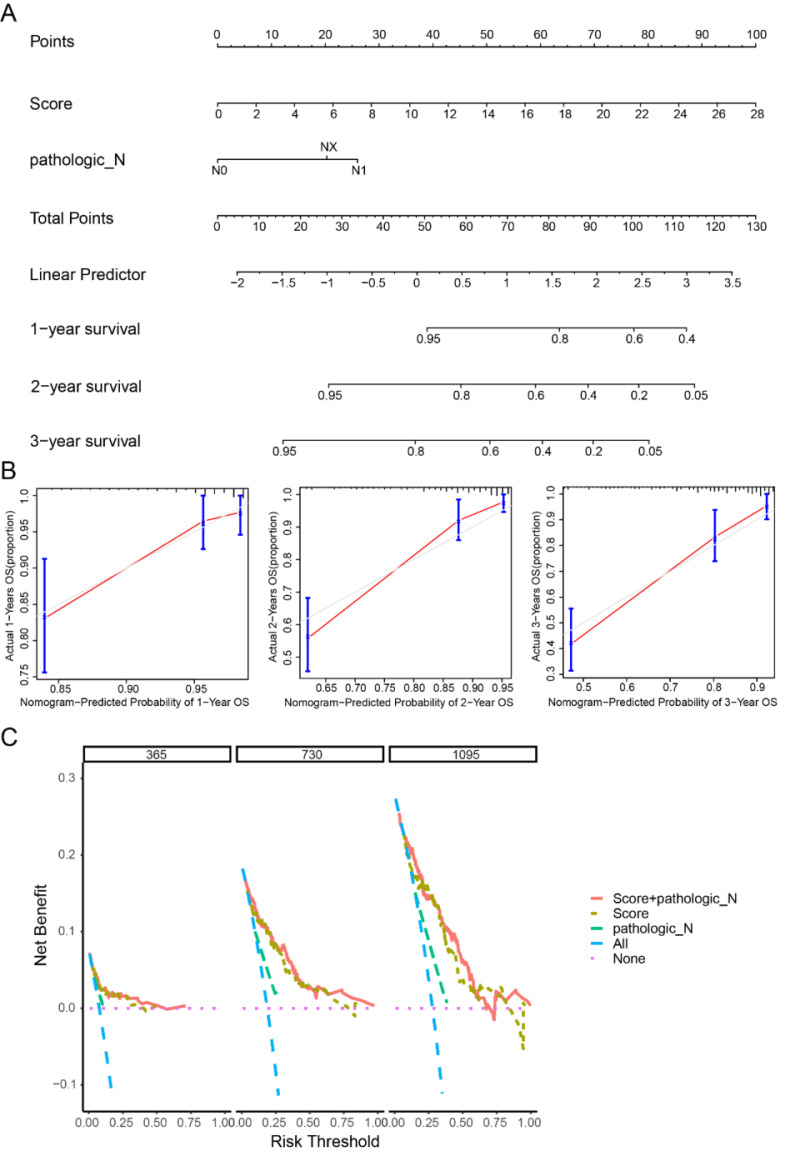
**Nomogram has a good prognostic ability for cervical cancer patients.** (A) Construct nomograms containing independent prognostic factors (N staging and score) using 'rms' packages. (B) Calibration curves for 1-year, 2-year, and 3-year overall survival were plotted using the 'rms' package to verify nomogram's predictive power. (C) 'ggDCA' package was used to construct DCA curves to evaluate the clinical application of nomogram.

In order to explore the correlation between the 33-lncRNA-CESC model and other clinical information, clinical information of CESC patients and the lncRNA expression level of the model were used to plot a heatmap. As shown in supplementary Figure S3, model risk score grouping was positively correlated with lncRNA expression level, but the correlation between risk score grouping and other clinical conditions was not obvious. Therefore, in order to explore whether the 33-lncRNA-CESC model was an independent prognostic factor for CESC patients, multi-factor Cox regression analysis was performed on the clinical information of CESC patients and the risk score of the 33-lncRNA-CESC model. The results revealed that the model score (p < 0.001; coefficient = 1.665) and N stage (p = 0.00825; coefficient = 2.717) were independent risk factors affecting the prognosis of CESC patients (supplementary Table S8). In order to translate 33-lncRNA-CESC model into clinical practice, a nomogram with independent risk factors (phase N, model score) was created. This nomogram can effectively estimate and quantify survival of CESC patients at 1 -, 2 -, and 3-year time points (Figure 3A). In order to verify the prediction capability of the nomogram, a calibration curve was drawn, and the predicted values were in good agreement with the actual values (Figure 3B). Finally, as shown in Figure 3C, DCA curves for years 1, 2, and 3 indicate that patients benefit from using this nomogram, further confirming the clinical applicability of the model-based nomogram to estimate OS in CESC patients.

### The 33-lncRNA-CESC model is associated with tumor immune microenvironment

**Figure 4:**
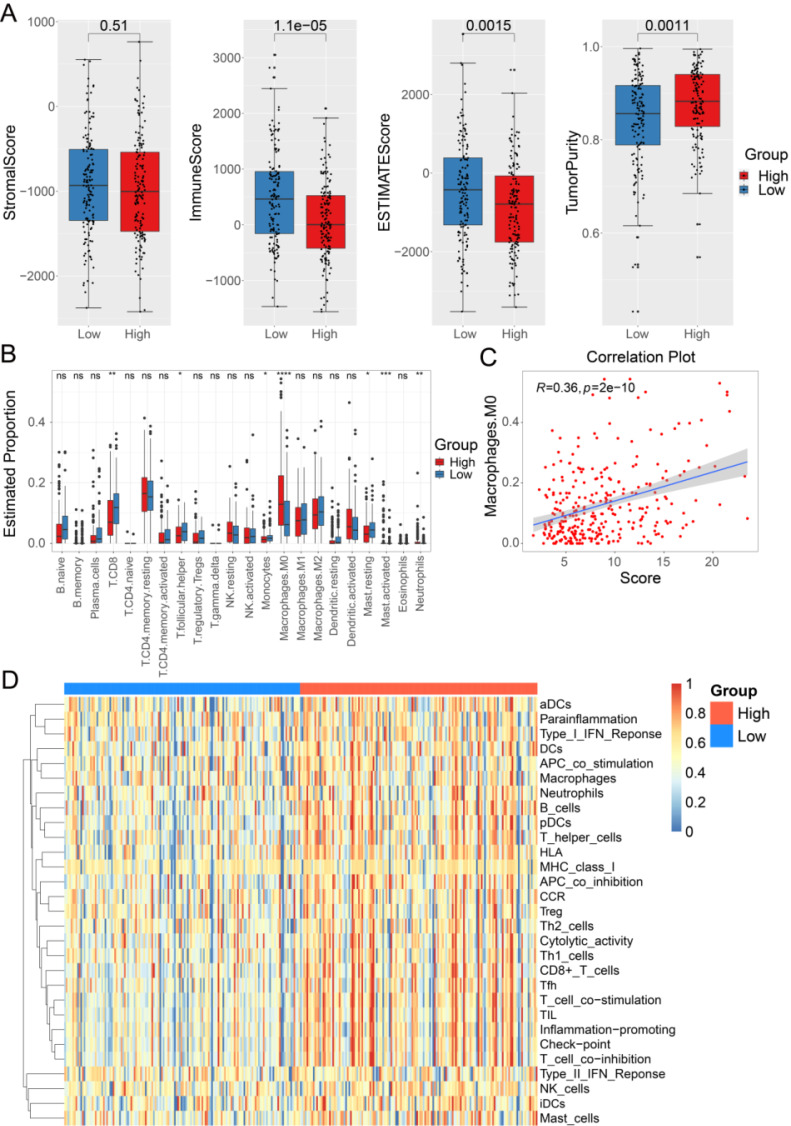
**The landscape of immune infiltration in the high-risk and low-risk groups.** (A) Immunological scores, stromal scores, estimative scores, and tumor purity were compared between low-risk (n=146) and high-risk (n=147) groups of a total of 293 CESC patients grouped by model risk scores. (B) 'CIBERSORT' package was used to analyze the difference of 22 kinds of immune cell infiltration between high-risk group and low-risk group. (C) 'Corrr' package was used to analyze the correlation between M0 macrophage infiltration level and model score. (D) The functional differences of immunity between low-risk and high-risk groups based on ssGSEA scores were analyzed using 'GSVA' package.

To further explore the relationship between the 33-lncRNA-CESC model and anti-tumor immunity in CESC patients, the 'estimate' package was used to measure the difference in stromal and immune cell infiltration between the low-risk and high-risk groups of 293 CESC patients with model risk scores, and the results showed that the high-risk group had lower sample immune and stromal scores and higher tumor purity (Figure 4A). To further understand the significant differences in TME between different risk groups, the 'CIBERSORT' package was used to calculate the relative expression levels of 22 common aggressive immune cells between the two groups. The results show that Macrophages M0, Mast cells activated, and Neutrophils are significantly upregulated in the high-risk group; in the low-risk subgroup, the level of T cells CD8, T cells follicular helper, Monocytes, and Mast cells resting is significantly upregulated (Figure 4B). Notably, the infiltration level of Macrophages M0 (Cor = 0.34, p < 0.0001) is positively correlated with the model score, suggesting that poor prognosis in the high-risk group may be related to an immunosuppressive microenvironment (Figure 4C). Additionally, ssGSEA analyses were performed using the 'GSVA' package to assess enrichment levels of 29 immune-related functions between the two subgroups. The ssGSEA analysis results confirm significant differences between the two subgroups, including APC co-stimulation, B cells, APC co-inhibition, CCR, CD8+ T cell checkpoint, cytolytic activity, DCs, mast cells, neutrophils, HLA, idc, macrophages, NK cells, T helper cells, pDCs, T cell co-inhibition, T cell co-stimulation, type 2 inflammation, Tfh, TIL, type II IFN response, type I IFN response, and Tregs (Figure 4D).

### The 33-lncRNA-CESC model plays a role in the progress of CESC through EGFR and other pathways

To elucidate the differences in enriched pathways between the low-risk and high-risk groups, Gene Set Enrichment Analysis (GSEA) was conducted using KEGG and Reactome pathway gene sets. The results revealed enrichment of pathways such as FGFR3(fibroblast growth factor receptor 3) mutant receptor activation, GRB2 events in EGFR signaling, PD-1 signaling, Signaling by FGFR3 point mutants in cancer, FRS-mediated FGFR1 signaling, Signaling by EGFR in Cancer, Downstream signaling of activated FGFR1, PERK regulates gene expression, MAP2K and MAPK activation, PI3K Cascade, Signaling by FGFR1, and Oncogenic MAPK signaling in the high-risk group (Figure S7). The analysis results indicate that lncRNAs in the 33-lncRNA-CESC model may affect CESC progression through these pathways. Previous studies have shown that the EGFR pathway is regulated by lncRNA FAM83H-AS1^25^, and lncRNA CAR10 can stably up-regulate EGFR by binding to transcription factor YB-1^26^. Knockdown of lncRNA RMEL3 and URHC can regulate cell proliferation and induce apoptosis through ERK/MAPK pathway inactivation^27^,^28^. lncRNA BANCR knockdown significantly inhibits ERK/MAPK pathway by decreasing MMP1 and MMP2^29^, and MALAT1 has been confirmed to activate PI3K/Akt pathway by increasing Akt phosphorylation^30^. These reports are consistent with our analysis. However, FGFR appears in the analysis as a new pathway, suggesting that this pathway may be a new research direction for the treatment of cervical cancer.

### The key lncRNA EGFR-AS1 was identified and significantly correlated with FAM83B

**Figure 5:**
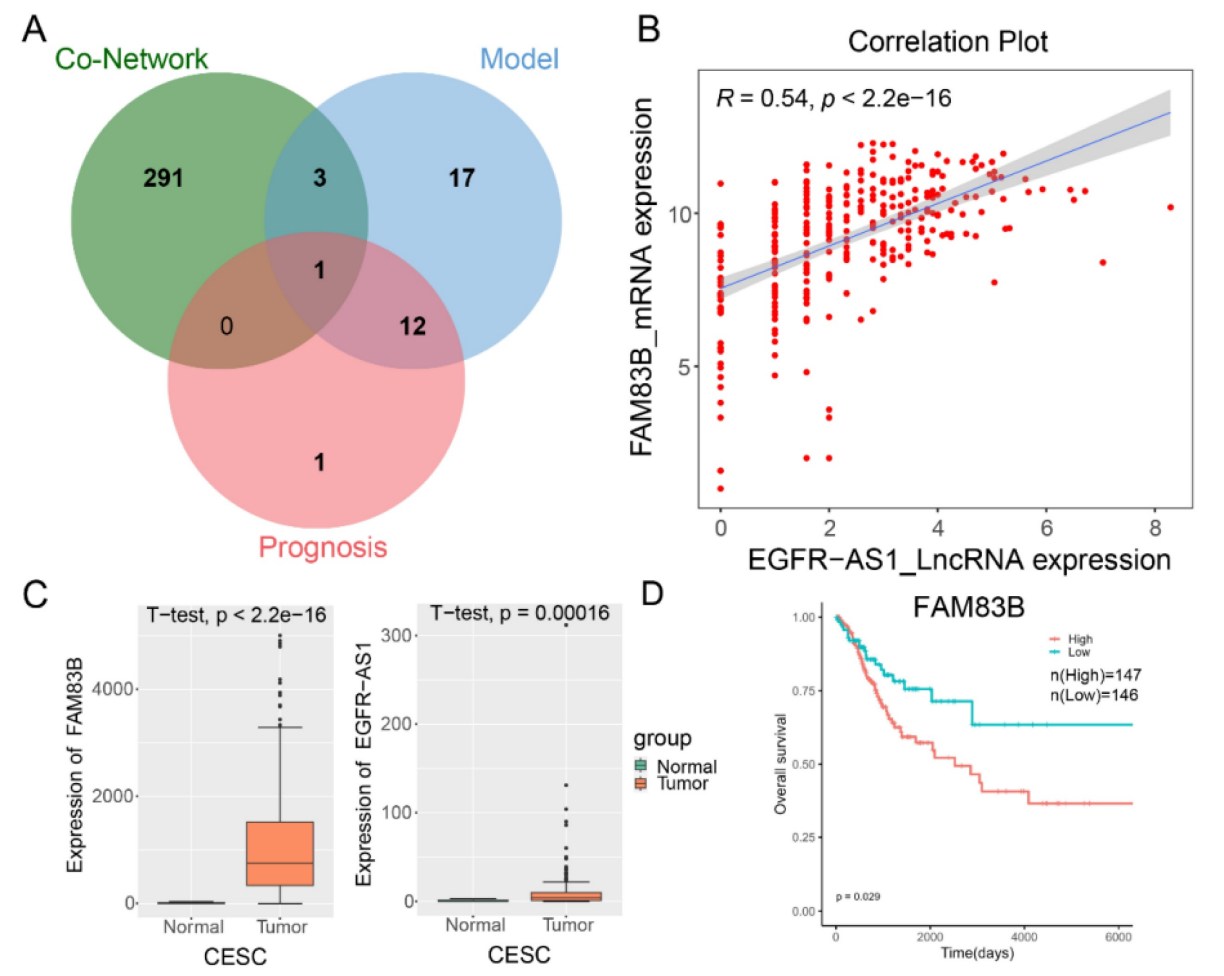
**The key lncRNA EGFR-AS1 was identified and significantly correlated with FAM83B.** (A) 'Venn' packages were used to map co-expression network genes, prognostic important genes, and internal the 33-lncRNA-CESC model's genes. (B) Using the transcriptome data of 306 CESC patients in the TCGA database, a scatter plot was drawn to show the correlation between the expression level of FAM83B and EGFR-AS1. (C) The expression of FAM83B and EGFR-AS1 in 306 CESC patients in the TCGA database. The gene expression levels are estimated and expressed as count. (D) Kaplan-Meier curve of FAM83B expression in TCGA-CESC patients were plotted using 'survival' package.

To explore the potential mechanisms of cervical cancer prognosis and treatment in more depth, we created a Venn diagram that intersects co-expression network genes, internal the 33-lncRNA-CESC model's genes, and prognostic important genes. The analysis showed that only lncRNA EGFR-AS1 existed in the cross-concentration, indicating that EGFR-AS1 was the only lncRNA in the 33-lncRNA-CESC model that could independently predict the prognosis of CESC, and FAM83B was the only gene related to EGFR-AS1 in the CESC co-expression network (Figure 5A and S4). Correlation analysis showed a strong positive correlation between them (Figure 5B), and their expression levels in cancer tissue samples were also significantly higher than that in adjacent normal tissue samples (Figure 5C). In addition, we explored the prognostic correlation between FAM83B and patients with cervical cancer in the TCGA cohort, and found that high expression of FAM83B was significantly correlated with poor prognosis in patients with cervical cancer (Figure 5D).

### EGFR-AS1/FAM83B participates in CESC progression through the EGFR signaling pathway

**Figure 6:**
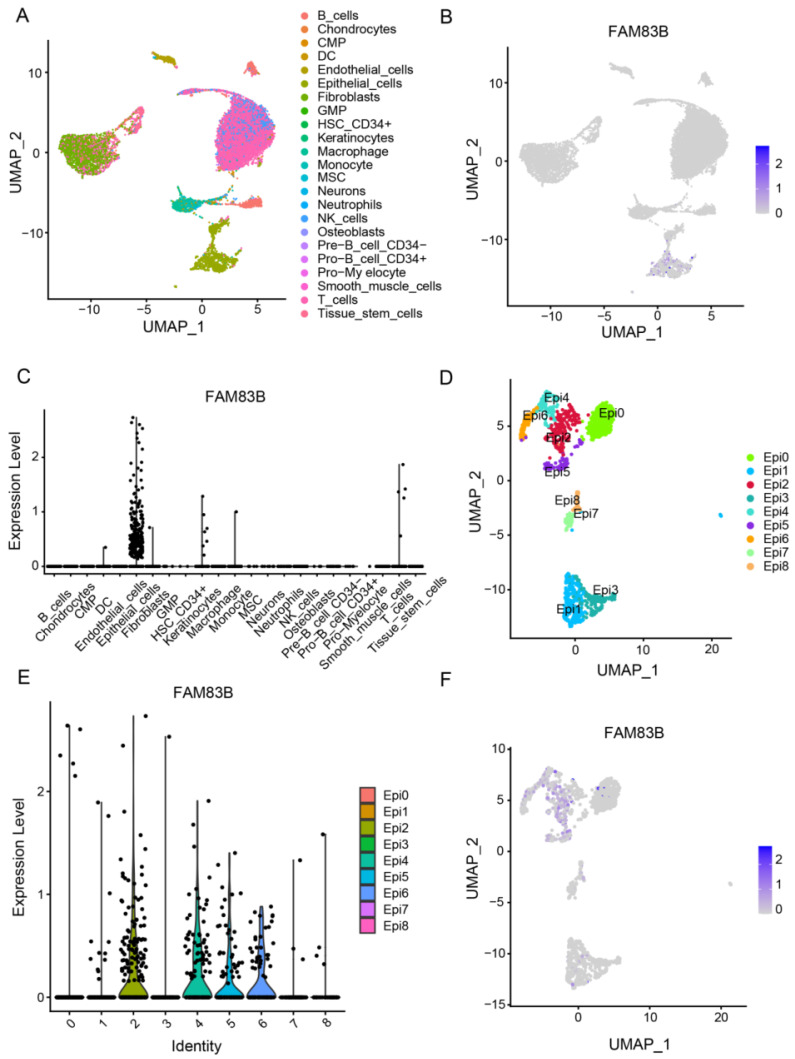
**FAM83B was clustered in cervical epithelial cells.** (A)UMAP dimensional-reduction clustering was performed on 13634 single-cell data using the Seurat package, and 23 cell types were annotated using the SingleR package. (B, C) The expression of FAM83B in 13634 single cell data was statistically analyzed by Seurat package. (D) UMAP subclustering was performed for all epithelial cells using Seurat packets. (E, F) Seurat packets were used to analyze the expression of FAM83B in epithelial cells.

In order to exclude the heterogeneity of batch sequencing and explore the mechanism of FAM83B/EGFR-AS1 in CESC progression, 16312 single-cell data were downloaded from GEO database, 13634 quality-controlled CESC cells were cluster annotated, and a total of 23 cell types were annotated (Figure 6A). Results revealed a high expression of FAM83B in epithelial cell types (Figures 6B, C). Consequently, further sub-clustering of epithelial cells was performed, resulting in the clustering of epithelial cells into 9 distinct cell clusters (Figure 6D). It was observed that FAM83B was predominantly highly expressed in cluster 2, 4, 5, and 6 (Figures 6E, F).

**Figure 7:**
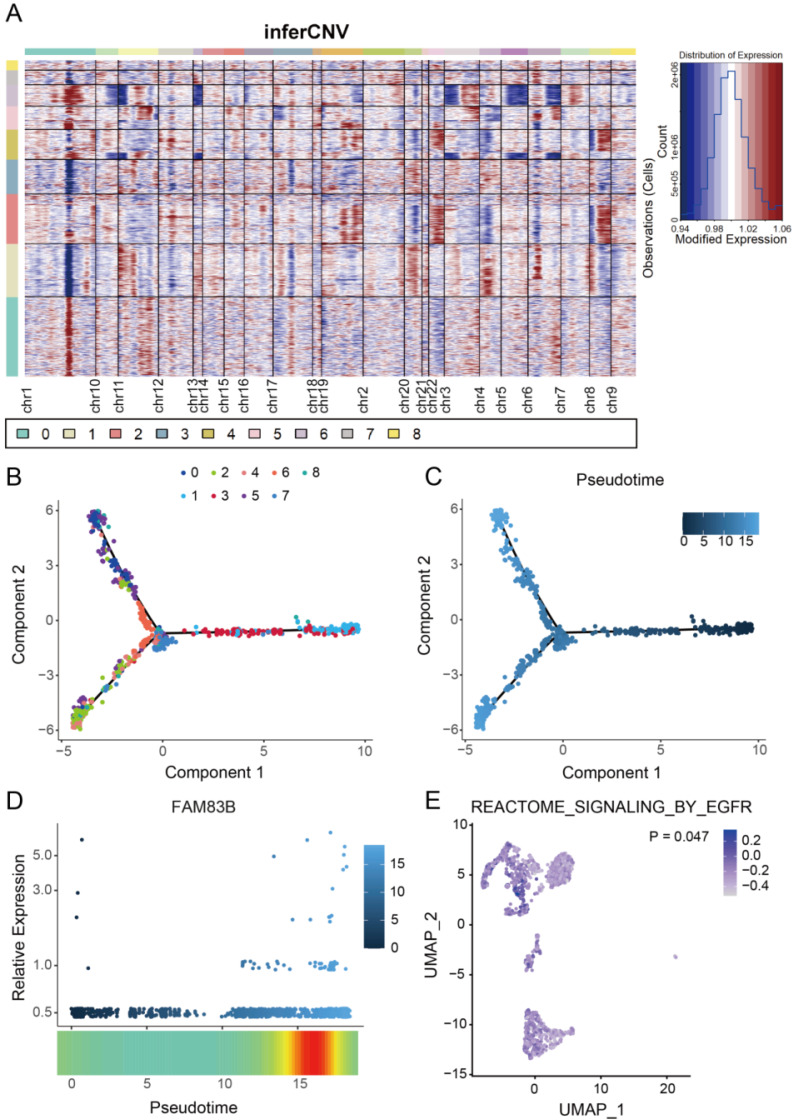
**FAM83B participates in CESC progression through the EGFR signaling pathway.** (A) Using the 'inferCNV' package to perform hierarchical clustering analysis on all epithelial cell clusters and creating a heatmap. (B, C) Using the 'monocle2' package to perform trajectory analysis on all epithelial cell clusters. Cells were color-coded by cluster(B) or pseudo-time(C). (D) The scatterplot of the expression level of FAM83B over time was plotted using the 'monocle2' package. (E) Cell enrichment to the EGFR pathway in epithelial cell clusters was mapped using 'GSVA' packages, where the color depth of the points indicates the degree of enrichment of the gene set.

The 'inferCNV' package was then used to distinguish tumor cells from normal epithelial cells. inferCNV analysis revealed a significant increase in the CNV levels within clusters 2, 4, 5, and 6 where FAM83B was located (Figure 7A), indicating that FAM83B is indeed highly correlated with CESC tumor cell. In order to determine the spatio-temporal position of FAM83B during the transition from normal cells to cancer cells, all epithelial cells were selected for cell trajectory analysis and pseudo-time analysis was performed based on Monocle2. The results showed that tumor differentiation presented a non-random expression pattern over time. FAM83B (clusters 2, 4, 5 and 6) is in the middle and late stages of the evolution of normal cells into tumor cells (Figure 7B,C). During this evolution, the expression of FAM83B gradually increased (Figure 7D). And GSVA results showed that the cell cluster in which FAM83B was located was involved in EGFR pathway (Figure 7E). Unfortunately, EGFR-AS1 was not included in this single cell dataset, but due to the high correlation between EGFR-AS1 and FAM83B, and the enrichment of the EGFR signaling pathway in the 33-lncRNA-CESC model's pathway analysis result, we speculate that EGFR-AS1 and FAM83B may jointly influence the occurrence and development of CESC through the EGFR signaling pathway.

### EGFR-AS1 and FAM83B promote the proliferation and migration of CESC cells, and regulate each other

**Figure 8:**
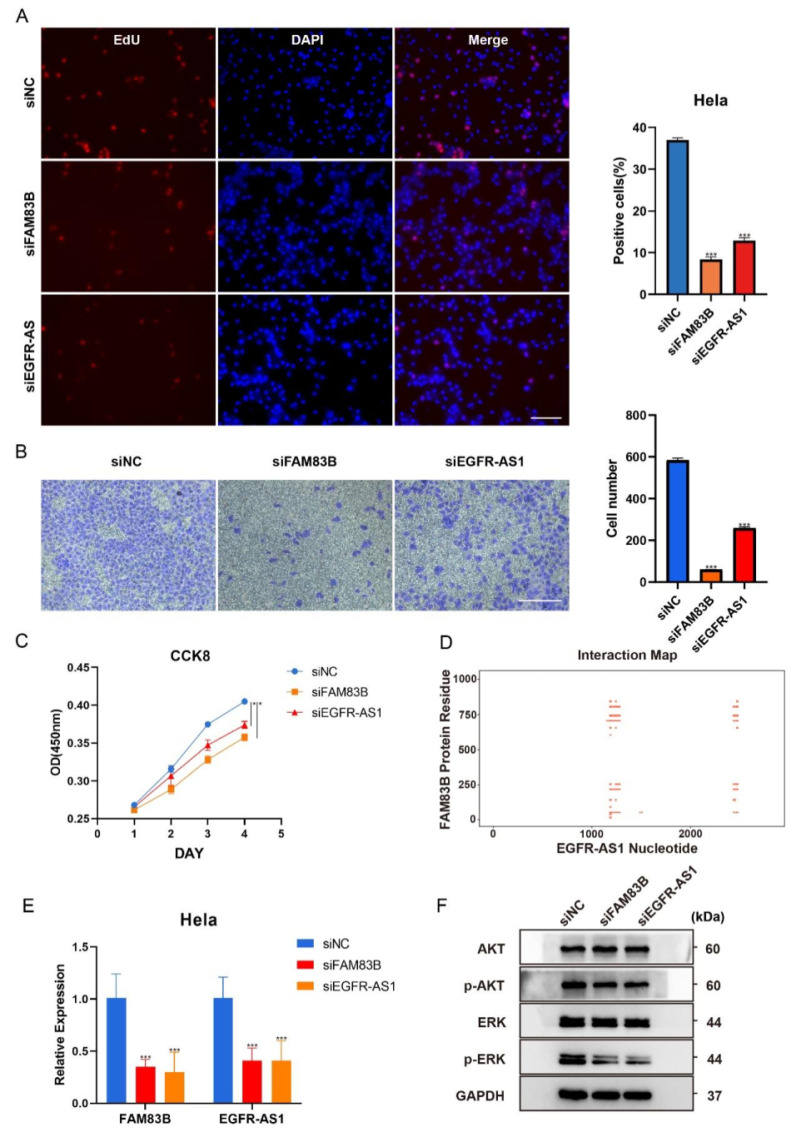
**EGFR-AS1 and FAM83B promote the proliferation and migration of CESC cells, and regulate each other.** HeLa cells were transfected with siNC, siFAM83B or siEGFR-AS1 for 24 h. (A) The EdU incorporation was quantitated at 24h. Scale bar, 100μm. (B) Quantitative analysis of cell migration assays. Migration was analyzed at 36 h, Scale bar, 100 μm. (C) The HeLa cell viability was determined by CCK8 kit for 1~4days. (D) Heatmap of RNA-protein interactions was generated using the catRAPID database, with the full length of FAM83B protein on the Y-axis and the full length of EGFR-AS1 nucleic acid on the X-axis. Red indicates areas of interaction. (E) qRT-PCR was used to verify the mRNA expression of FAM83B and EGFR-AS1 after gene knockdown. (F) The protein levels of ERK, phosphorylated ERK, AKT and phosphorylated AKT in HeLa cells were analyzed by Western blotting. In order to improve the clarity and simplicity of the report, the gel has been cropped and the original document can be found in supplementary document figure S9. Data represent the mean ± SD. All experiments were performed in triplicates. *, *P*<0.05, **, *P* < 0.01, ***, *P*< 0.001.

To further understand the role and relationship between FAM83B and EGFR-AS1 in CESC, transient knockdown of FAM83B and EGFR-AS1 was performed in HeLa cells. EdU labeling analysis indicated that knockdown of FAM83B or EGFR-AS1 significantly inhibited the proliferative and migration capacity of cervical cancer cells (Figure 8A-C), indicating that EGFR-AS1 and FAM83B play an important role in maintaining malignant manifestations of cervical cancer cells. Analysis using RIsearch2 indicated potential interactions between FAM83B and EGFR-AS1 at 7 sequence positions (Figure S8), while catRAPID analysis suggested the presence of interacting domains between EGFR-AS1 and the protein translated by FAM83B (Figure 8D). This suggests that EGFR-AS1 and FAM83B may interact at either the transcriptional level or the protein level. In order to further verify whether EGFR-AS1 and FAM83B can regulate each other, qRT-PCR was used for verification. The result showed that knocking down FAM83B significantly inhibited the expression of EGFR-AS1, similarly, knocking down EGFR-AS1 significantly inhibited the expression of FAM83B, suggesting that there may be a synergistic effect between them at the transcriptional level (Figure 8E). In addition, according to the analysis results, it was speculated that EGFR-AS1 and FAM83B might regulate the EGFR pathway. In order to confirm this hypothesis, EGFR-AS1 or FAM83B were knocked down in HeLa cells, and the expression levels of downstream of EGFR signaling pathway such as phosphorylated ERK and phosphorylated AKT were detected. The results showed that the levels of phosphorylated ERK and phosphorylated AKT in HeLa cells with EGFR-AS1 or FAM83B knocked down were inhibited, indicating that EGFR-AS1 and FAM83B can indeed regulate the EGFR pathway in cervical cancer cells (Figure 8F). These results indicate that EGFR-AS1 can interact with FAM83B and promote the proliferation and migration of cervical cancer cells through EGFR signaling pathway.

## Discussion

In this study, a CESC mRNA-lncRNA co-expression network was constructed, and then a CESC-related prognostic model was obtained by using the lncRNA in the network, which could significantly predict the prognosis of CESC patients and was correlated with the tumor microenvironment. Then a nomogram was constructed using the calculated risk score and other clinical information to verify the clinical applicability of this nomogram. We found that EGFR-AS1 was the only lncRNA in the 33-lncRNA-CESC model that could independently predict the prognosis of CESC, and FAM83B was the only gene associated with EGFR-AS1 in the CESC co-expression network. Single-cell data analysis showed that EGFR-AS1 and FAM83B may influence CESC prognosis through EGFR signaling pathway. The experimental results showed that both EGFR-AS1 and FAM83B could affect the proliferation and migration of CESC cells, and there was a regulatory relationship between them at the transcriptional level.

Multivariate Cox regression and random forest are both statistical methods used to construct tumor prognosis models, and they have their own advantages and disadvantages. The advantage of multi-factor Cox regression is that it can provide interpretable results, and the coefficient estimated by Cox regression can be used to estimate the risk ratio, which indicates the relative risk of events occurring between two groups with different levels of predictors 31. However, Cox regression also has shortcomings. For example, Cox regression assumes that the influence of the predictors is linear, which means that there is no interaction between the predictors. However, as we all know, there are complex regulatory relationships between genes, and many genes are not linear regulatory relationships, so using Cox regression to predict factors with mutual regulation may be inaccurate. So to solve these problems, consider using machine learning algorithms, such as random forests or support vector machines, that are not affected by the assumptions of traditional survival analysis techniques. Random forest is the most representative Bagging algorithm in ensemble learning. Compared with Cox regression, its advantage is that it can capture complex interactions between variables, including nonlinear relationships, so it is very suitable for the same complex transcriptional regulation process 32. In addition, the random forest algorithm excels at handling high-dimensional data, meaning it can efficiently process a vast number of variables simultaneously, making it highly suitable for the contemporary big data landscape. Moreover, the random forest does not make any assumptions about the underlying distribution of the data or the relationship between variables 32. When modeling data using statistical methods, we frequently make assumptions regarding the data's underlying distribution and the interrelationships among variables. In contrast, the random forest constructs its model by iteratively dividing the data into subsets based on the values of the predictor variables. This approach enables it to capture intricate interactions and nonlinear relationships without the need to presume a specific form for these relationships 33. Consequently, the random forest is particularly advantageous when the underlying relationship between variables is poorly understood or highly nonlinear. We compared our 33-lncRNA-CESC model with four cervical cancer prognosis models constructed using Cox regression in other literature 8,34-36. ROC curve results (supplemental Figure S9) showed that the AUC value of 33-lncRNA-CESC model was the largest (AUC=0.787,0.83,0.788) regardless of whether the prognosis of cervical cancer patients was evaluated for 1 year, 2 years or 3 years. This may indicate that the ensemble learning approach we used may be more suitable for high-dimensional data. In summary, the choice of method for constructing the model should depend on the specific research question, the nature of the data, and the tradeoff between interpretability and predictive accuracy. Therefore, for prognostic studies, combining the interaction between predictors and using alternative analytical methods may be two potential avenues for future development.

Prognostic models containing 33 lncRNAs were constructed, some of which have been reported to exist in CESC or other cancers. The 8-lncRNA model including LINC02802 can predict the prognosis of CESC and contribute to determining immunotherapy strategies^37^. The feedback loop involving DDN-AS1-miR-15a/16-TCF3 is involved in the proliferation, migration, and invasion of cervical cancer cells^38^. LINC02783 is highly expressed in RCC patients and indicates a poor prognosis. LINC02783 can affect the occurrence and progression of RCC through the miR-20b/Sox-4 axis, making it a promising target for the treatment of RCC^23^. SPRY4-AS1 is considered as a novel therapeutic target for the treatment of hepatocellular carcinoma^24^. The ITGB1-DT/ARNTL2 axis may serve as a novel biomarker for lung adenocarcinoma^39^. Knocking down LINC02015 can promote cell proliferation and inhibit apoptosis of HAVSMCs through the RXRA-related transcriptional regulatory network, providing a potential therapeutic target for aortic diseases^40^. LINC00460 promotes cell migration and invasion in lung cancer by inducing epithelial-mesenchymal transition (EMT) in cancer cells^41^. LINC01615 maintains cell survival under nutrient starvation by modulating the pentose phosphate pathway and regulates chemosensitivity in colorectal cancer. LINC01615 knockdown combined with oxaliplatin achieved remarkable antitumor effects in PDO and PDX models, which provides a potential therapeutic target for CRC^42^. The expression levels of FLNB-AS1 are positively correlated with the survival probability of breast cancer patients^43^. A total of 10 lncRNAs have been reported, while another 23 have been newly identified. To investigate genes associated with mutations in CESC, mutation data from 289 CESC cases were utilized for single nucleotide mutation and copy number variation analysis (Supplementary Figure 4-5). However, no genes in the co-expression network were found in the mutation results, so no follow-up studies were conducted.

We also found through GSEA analysis of the 33-lncRNA-CESC model are mainly involved in FGFR3 mutant receptor activation, EGFR signaling in GRB2 events, FGFR3 point mutant signaling in cancer, FRS-mediated FGFR1 signaling, EGFR signaling in cancer, downstream signaling in activating FGFR1, and MAP2K and MAPK activation, FGFR1 signaling, Oncogenic MAPK signaling and other signaling pathways. At present, the role and mechanism of lncRNA in some signaling pathways of cervical cancer have been reported, such as Wnt signaling pathway EGFR signaling pathway, ERK/MAPK signaling pathway, etc.^44^. However, the FGFR family-related gene set enriched by GSEA in this study has not been reported. The FGFR family consists of four members: FGFR1, FGFR2, FGFR3, and FGFR4^45^. These receptors are tyrosine kinase receptors that play vital roles in various cellular processes including cell proliferation, differentiation, migration, and survival. They are activated by binding to fibroblast growth factors (FGFs), a large family of polypeptide growth factors. FGFR1, FGFR2, and FGFR3 are known to have multiple isoforms generated through alternative splicing, each with distinct tissue distributions and functions^46^. FGFR4, however, does not undergo extensive alternative splicing^47^. Upon ligand binding, FGFRs dimerize, leading to auto-phosphorylation of tyrosine residues in their cytoplasmic domains, which in turn initiates downstream signaling cascades such as the MAPK, PI3K/AKT, and PLCγ pathways^48^,^49^. Dysregulation of FGFR signaling, either through overexpression, gene amplification, or activating mutations, has been implicated in various human diseases, including cancer and developmental disorders^50^. Therefore, targeting the FGFR signaling pathway may be a promising therapeutic strategy for the treatment of cervical cancer containing FGFR abnormalities.

EGFR-AS1 is a lncRNA that is transcribed from the antisense strand of the EGFR gene, which is the only lncRNA in the 33-lncRNA-CESC model that can be used as an independent prognostic indicator and exists in the CESC co-expression network. It has been implicated in various cellular processes and diseases, particularly cancer^51^. Studies have shown that EGFR-AS1 regulates the expression of EGFR mRNA by affecting its stability, thus affecting cervical cancer cell growth, proliferation and migration^6^. But, little is known about the regulatory effects of EGFR-AS1 with other genes. FAM83B, as the only gene associated with EGFR-AS1 in the CESC co-expression network, encodes a protein involved in activating EGFR binding activity, phosphatidylinositol 3-kinase (PI3K) binding activity, and protein kinase binding activity^52^. It participates in cell proliferation and the EGFR signaling pathway^53^, which is consistent with the results of our single cell analysis. FAM83B has also been reported to overactivate EGFR and downstream effector phospholipase D1^54^. This study confirms the potential regulatory relationship between EGFR-AS1 and FAM83B. However, the specific mechanism of their interaction remains unknown and warrants further investigation.

In conclusion, our study findings demonstrate that the 33-lncRNA-CESC model holds promise for assessing the prognosis and molecular characteristics of CESC patients, thus improving treatment strategies with potential clinical applications. Furthermore, our results indicate EGFR-AS1/FAM83B can promote the proliferation and migration of cervical cancer cells by regulating the EGFR signaling pathway.

## Supplementary Material

Supplementary figures and tables 2-8.

Supplementary table 1.
