# Supplementary material for: Construction of a Prognostic Model for Cervical Cancer Related to lncRNA Based on Differential Co-expression Network and Functional Study of Key Gene EGFR-AS1
Source: J Cancer. 2025 Mar 31;16(7):2321–38. doi: 10.7150/jca.108429 (PMC12036091; doi:10.7150/jca.108429)
Supplement: Supplementary file 1 — Supplementary figures and tables 2-8. [file jcav16p2321s1.pdf]

Figure S1. This process reveals a lncRNA model for predicting the prognosis of cervical cancer

Figure S2. PPI networks reveal protein interactions in cervical cancer. The protein-protein interaction network of 268 differential genes of CESC was analyzed using string database.

Figure S3. The overview of clinical information and model lncRNA expression levels for CESC patients. Clinical information and lncRNA expression heatmap of 293 CESC patients were drawn using ComplexHeatmap and circlize packages in R software.

Figure S4. Overall mutations in patients with TCGA-CESC. (A) The maftools package was used to generate chromosome mutation locus maps from 289 CESC patients. (B) The maftools package was used to generate single nucleotide mutation statistics for 289 CESC patients.

Figure S5. Top-ranking CNVS and SNPS in patients with TCGA-CESC. The maftools package was used to generate Mutation landscape of 289 CESC patients. Waterfall plot showing the mutation information of top20 genes with mutations.

Figure S6. 14 lncRNAs with significant prognosis were identified in CESC patients. According to the median expression level of lncRNA, they were divided into high and low groups. Then Kaplan-Meier curves were drawn for 14 lncRNAs with significant prognosis in TCGA-CESC patients using survival package.

Figure S7. Gene set enrichment analysis between low-risk group and high-risk group

Figure S8. RIsearch2 analysis indicated potential interactions between FAM83B and EGFR-AS1 at 7 sequence positions. Each column represents the ID of the query sequence, the start position of the query sequence, the end position of the query sequence, the ID of the target sequence, the start position of the target sequence, the end position of the target sequence, the chain of the interaction, and the free energy of the interaction, respectively (":"between GU," | "between CG and AU pairs).

Figure S9. ROC curves of several models

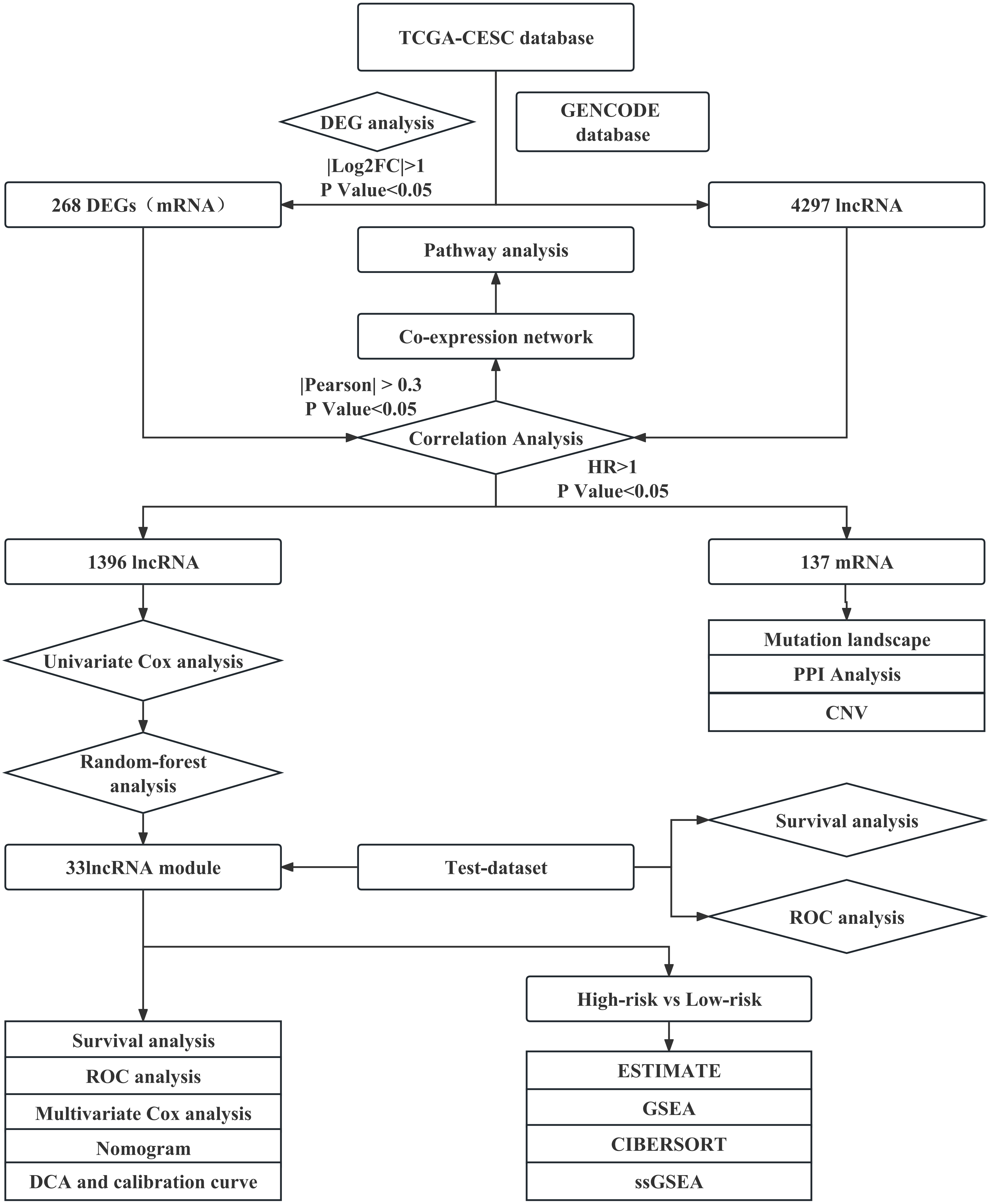
TCGA-CESC database
DEG analysis
GENCODE database
|Log2FC|>1
P Value<0.05
268 DEGs（mRNA）
4297 lncRNA
Pathway analysis
Co-expression network
|Pearson| > 0.3
P Value<0.05
Correlation Analysis
HR>1
P Value<0.05
1396 lncRNA
137 mRNA
Mutation landscape
PPI Analysis
CNV
Univariate Cox analysis
Random-forest analysis
Survival analysis
33lncRNA module
Test-dataset
ROC analysis
High-risk vs Low-risk
Survival analysis
ROC analysis
Multivariate Cox analysis
Nomogram
DCA and calibration curve
ESTIMATE
GSEA
CIBERSORT
ssGSEA

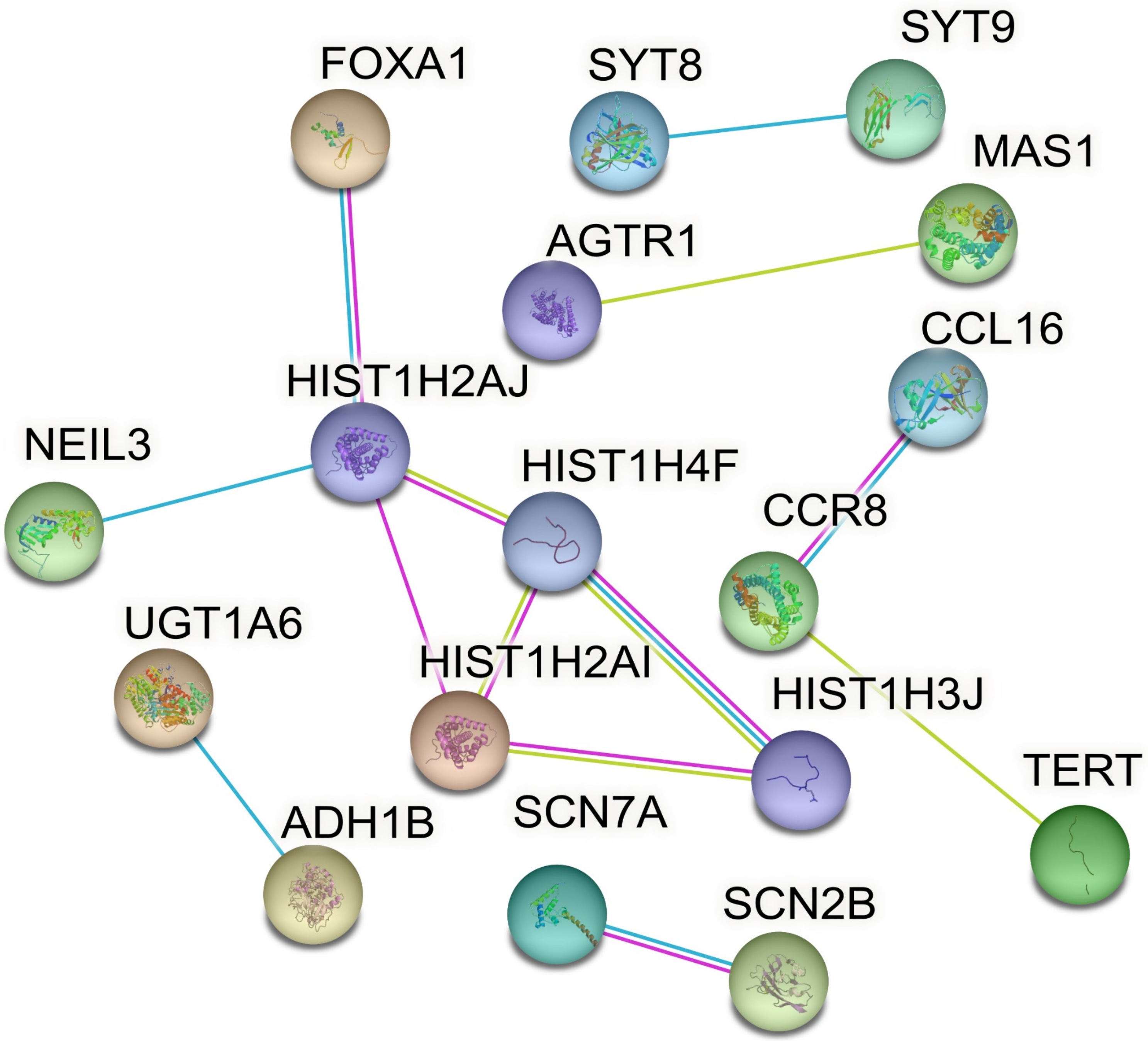
FOXA1
SYT8
SYT9
MAS1
AGTR1
CCL16
HIST1H2AJ
NEIL3
HIST1H4F
CCR8
UGT1A6
HIST1H2AI
HIST1H3J
ADH1B
SCN7A
TERT
SCN2B

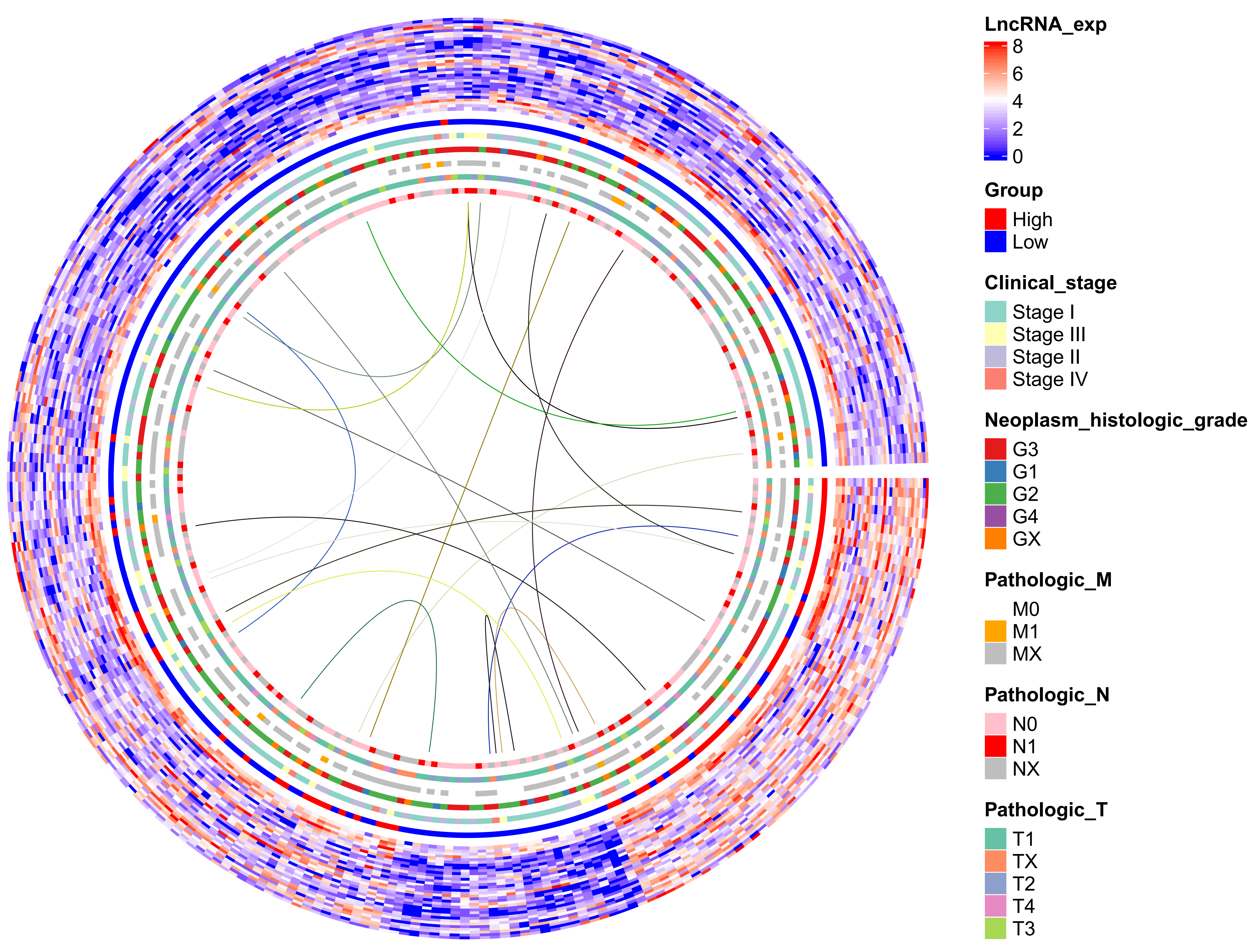
LncRNA_exp
8
6
4
2
0
Group
High
Low
Clinical_stage
Stage I
Stage III
Stage II
Stage IV
Neoplasm_histologic_grade
G3
G1
G2
G4
GX
Pathologic_M
M0
M1
MX
Pathologic_N
N0
N1
NX
Pathologic_T
T1
TX
T2
T4
T3

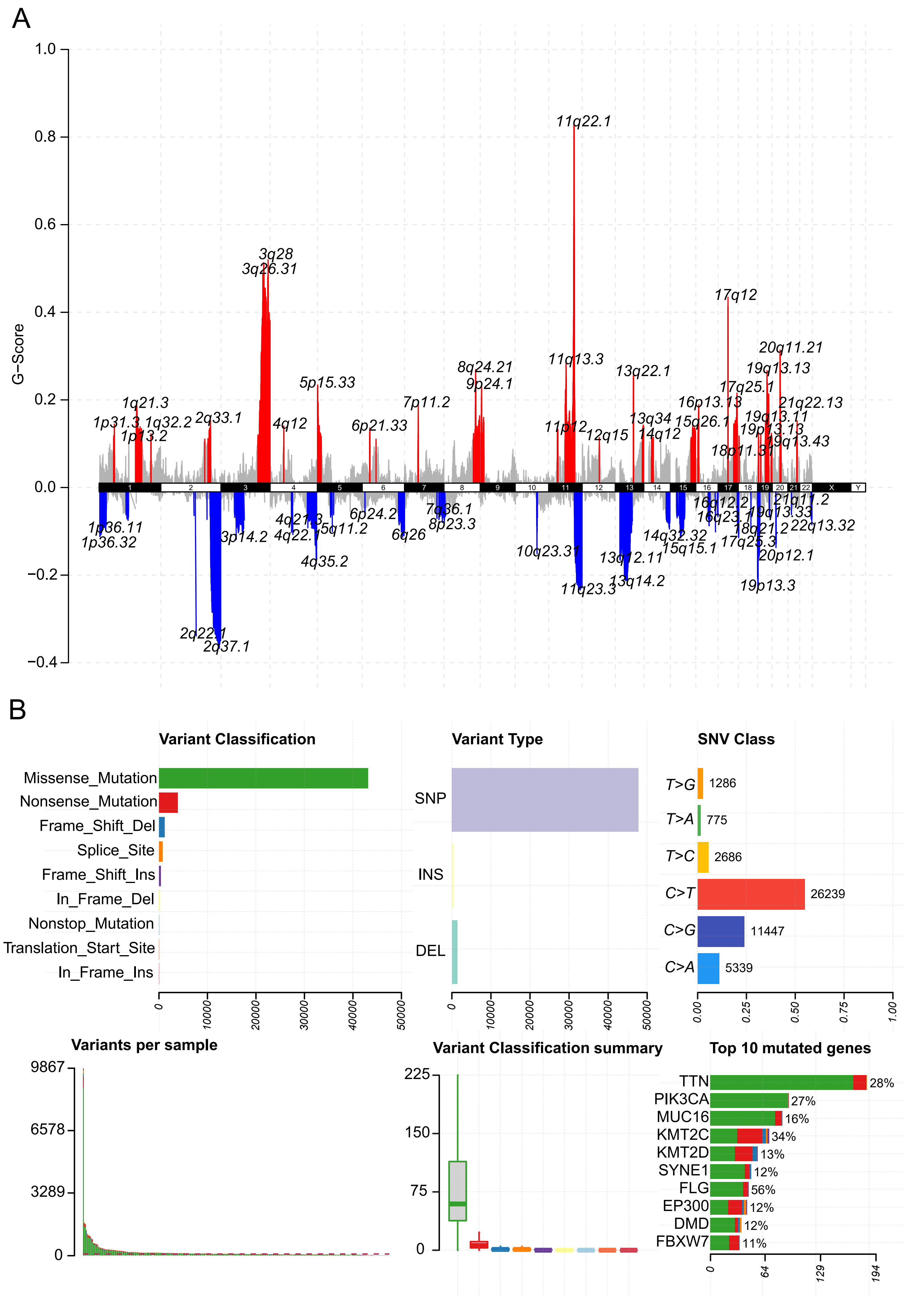

A
G−Score
1.0
0.8
0.6
0.4
0.2
0.0
−0.2
−0.4
11q22.1
3q28
3q26.31
17q12
20q11.21
19q13.13
11q13.3
8q24.21
9p24.1
13q22.1
5p15.33
17q25.1
1q21.3
7p11.2
16p13.13
21q22.13
2q33.1
1p31.3
1q32.2
4q12
6p21.33
13q34
15q26.1
19q13.11
1p13.2
11p12
12q15
14q12
19p13.13
19q13.43
18p11.31
16q12.2
21q11.2
16q23.1
19q13.33
7q36.1
22q13.32
1p36.11
4q21.3
6p24.2
8p23.3
18q21.2
1p36.32
3p14.2
4q22.1
5q11.2
6q26
14q32.32
17q25.3
15q15.1
10q23.31
13q12.11
20p12.1
4q35.2
11q23.3
13q14.2
19p13.3
2q22.1
2q37.1
B
Variant Classification
Missense_Mutation
Nonsense_Mutation
Frame_Shift_Del
Splice_Site
Frame_Shift_Ins
In_Frame_Del
Nonstop_Mutation
Translation_Start_Site
In_Frame_Ins
Variant Type
SNP
INS
DEL
SNV Class
T>G 1286
T>A 775
T>C 2686
C>T 26239
C>G 11447
C>A 5339
Variants per sample
9867
6578
3289
0
Variant Classification summary
225
150
75
0
Top 10 mutated genes
TTN 28%
PIK3CA 27%
MUC16 16%
KMT2C 34%
KMT2D 13%
SYNE1 12%
FLG 56%
EP300 12%
DMD 12%
FBXW7 11%

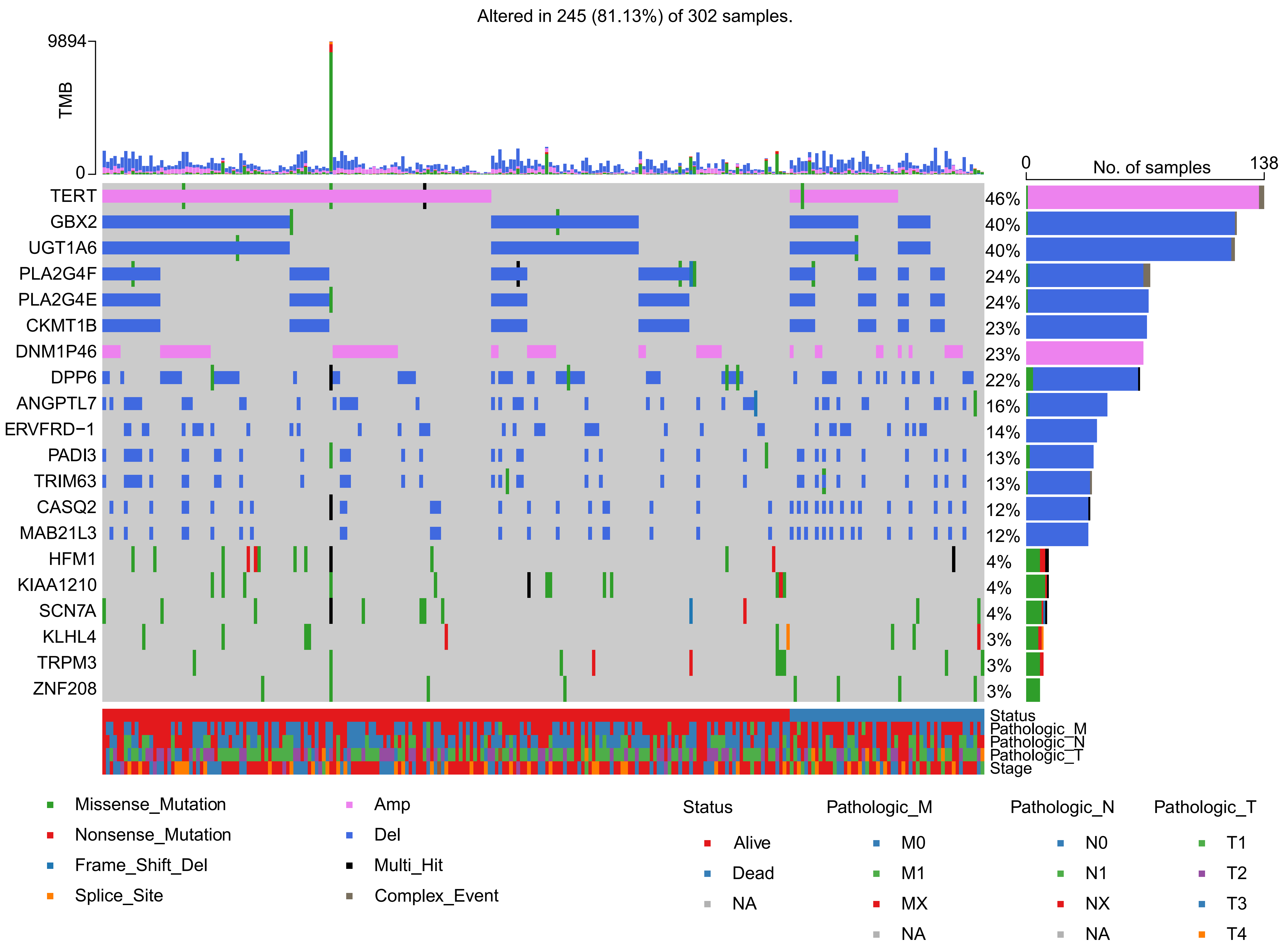

Altered in 245 (81.13%) of 302 samples.
9894
TMB
0
No. of samples
0
138
TERT 46%
GBX2 40%
UGT1A6 40%
PLA2G4F 24%
PLA2G4E 24%
CKMT1B 23%
DNM1P46 23%
DPP6 22%
ANGPTL7 16%
ERVFRD−1 14%
PADI3 13%
TRIM63 13%
CASQ2 12%
MAB21L3 12%
HFM1 4%
KIAA1210 4%
SCN7A 4%
KLHL4 3%
TRPM3 3%
ZNF208 3%
Status
Pathologic_M
Pathologic_N
Pathologic_T
Stage
Missense_Mutation
Nonsense_Mutation
Frame_Shift_Del
Splice_Site
Amp
Del
Multi_Hit
Complex_Event
Status
Alive
Dead
NA
Pathologic_M
M0
M1
MX
NA
Pathologic_N
N0
N1
NX
NA
Pathologic_T
T1
T2
T3
T4

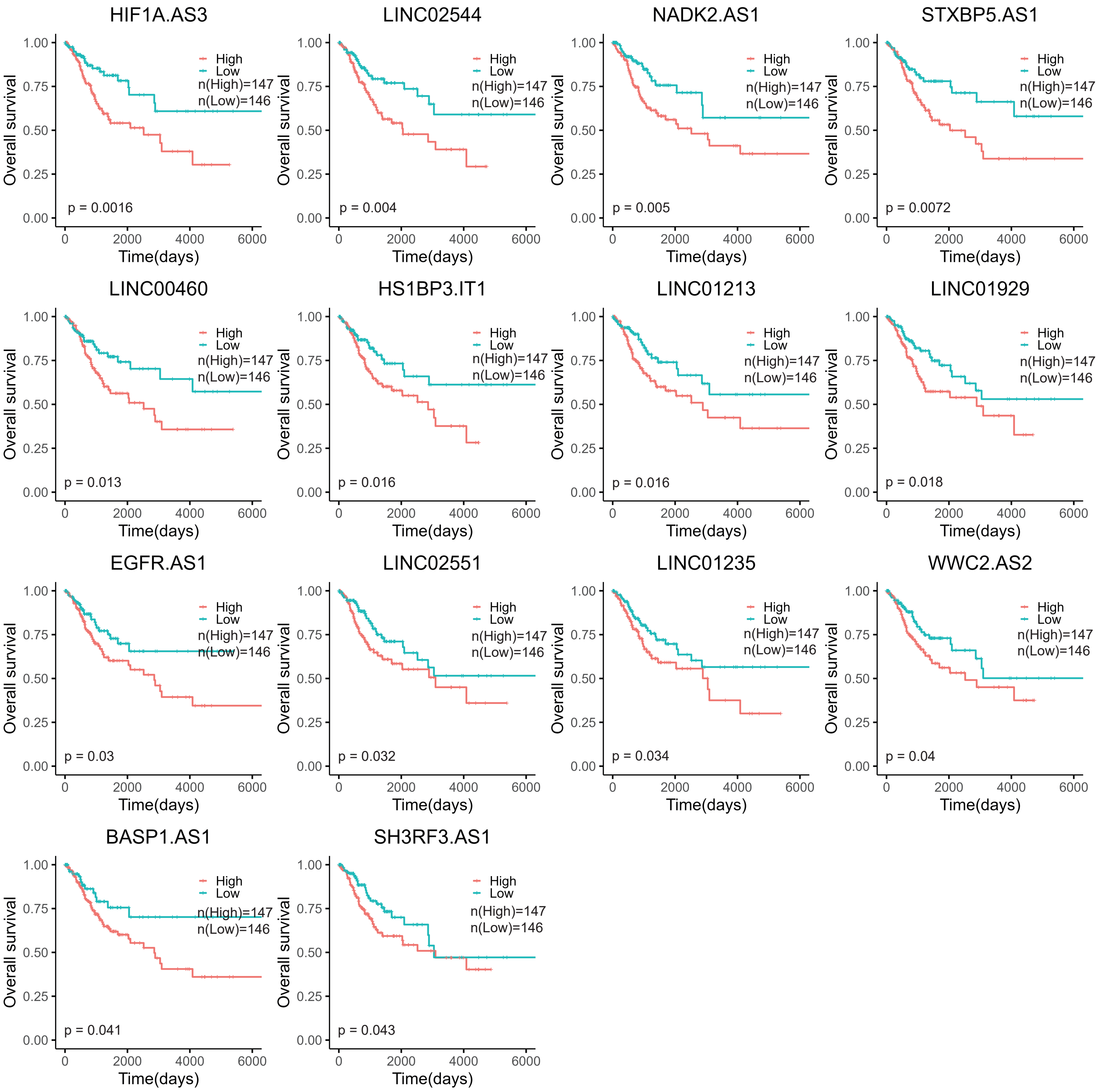

HIF1A.AS3
High
Low
n(High)=147
n(Low)=146
p = 0.0016
LINC02544
High
Low
n(High)=147
n(Low)=146
p = 0.004
NADK2.AS1
High
Low
n(High)=147
n(Low)=146
p = 0.005
STXBP5.AS1
High
Low
n(High)=147
n(Low)=146
p = 0.0072
LINC00460
High
Low
n(High)=147
n(Low)=146
p = 0.013
HS1BP3.IT1
High
Low
n(High)=147
n(Low)=146
p = 0.016
LINC01213
High
Low
n(High)=147
n(Low)=146
p = 0.016
LINC01929
High
Low
n(High)=147
n(Low)=146
p = 0.018
EGFR.AS1
High
Low
n(High)=147
n(Low)=146
p = 0.03
LINC02551
High
Low
n(High)=147
n(Low)=146
p = 0.032
LINC01235
High
Low
n(High)=147
n(Low)=146
p = 0.034
WWC2.AS2
High
Low
n(High)=147
n(Low)=146
p = 0.04
BASP1.AS1
High
Low
n(High)=147
n(Low)=146
p = 0.041
SH3RF3.AS1
High
Low
n(High)=147
n(Low)=146
p = 0.043
Overall survival
0.00
0.25
0.50
0.75
1.00
Time(days)
0
2000
4000
6000

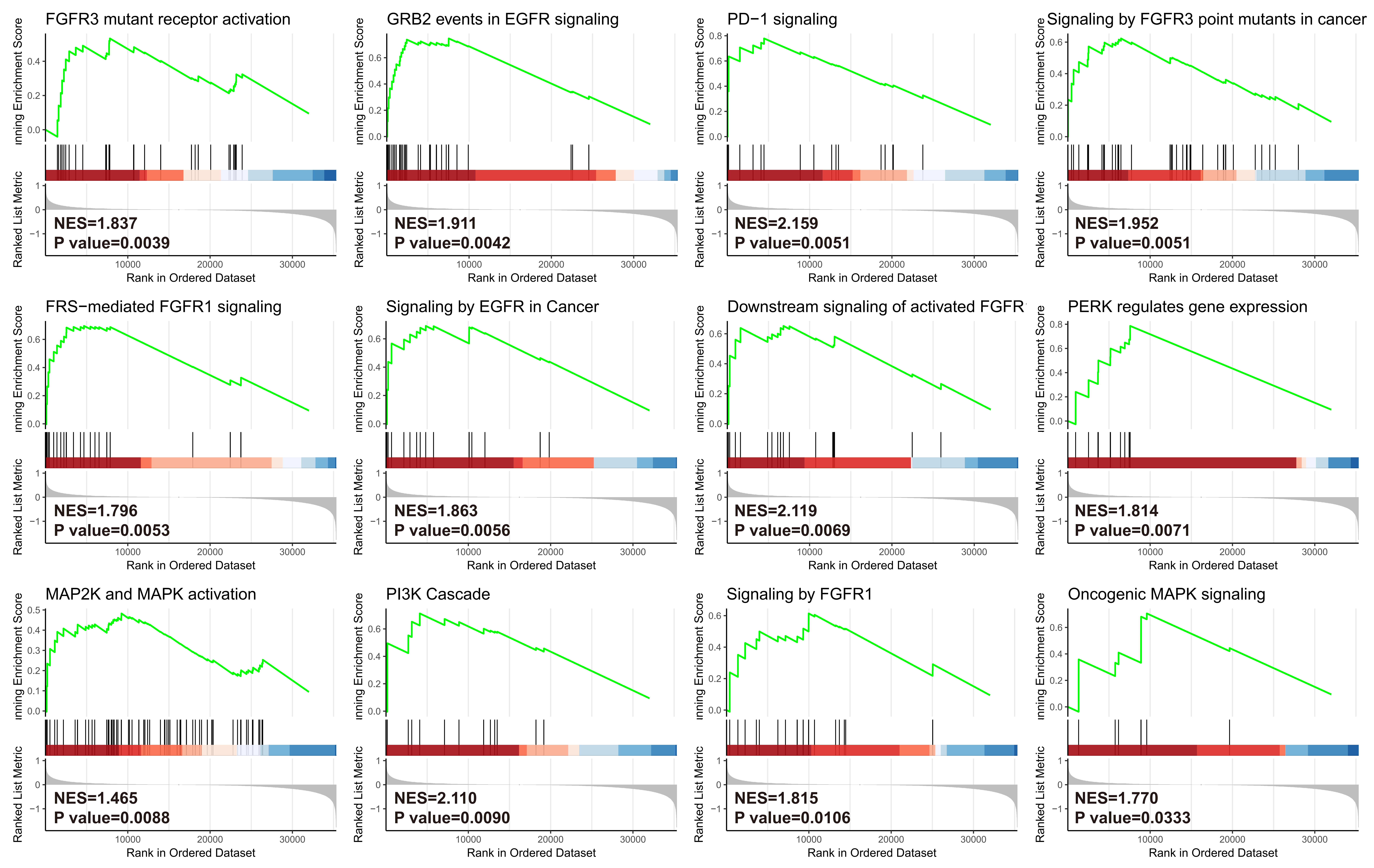

FGFR3 mutant receptor activation
NES=1.837
P value=0.0039
GRB2 events in EGFR signaling
NES=1.911
P value=0.0042
PD−1 signaling
NES=2.159
P value=0.0051
Signaling by FGFR3 point mutants in cancer
NES=1.952
P value=0.0051
FRS−mediated FGFR1 signaling
NES=1.796
P value=0.0053
Signaling by EGFR in Cancer
NES=1.863
P value=0.0056
Downstream signaling of activated FGFR
NES=2.119
P value=0.0069
PERK regulates gene expression
NES=1.814
P value=0.0071
MAP2K and MAPK activation
NES=1.465
P value=0.0088
PI3K Cascade
NES=2.110
P value=0.0090
Signaling by FGFR1
NES=1.815
P value=0.0106
Oncogenic MAPK signaling
NES=1.770
P value=0.0333
Running Enrichment Score
Ranked List Metric
Rank in Ordered Dataset

```
UCAAAGGUAGCUGAUUGAUGAGAGUUUCC---ACAUG--CAGAUGGG
     ||:|| ::|:|:|::|:|:||||     |||      ||||:
-----ccgucaguugauuguuuuaaagacaagguaaaaagguaccu
47551.1     864     909     NM_001010872.3  784     829     +       -2
CACCU-UUUACAUCUGCCUGGAGUU--------GGGUGAGCGC
||||    :|||:|||||::|||||         ||:  ||||
gugguguuguguggacgggucucaaaacgguuagccgaacgcg
47551.1     92      129     NM_001010872.3  1418    1464    -       -4
CAAGACUGGAUGGAGAUGGGAGGGG-----UUUGGGCAAAAGCAG
|   ||: :||||:|:||:::||::       |:::||       |||
gaggugg-ucuacuuuuauuuucuuguggaagaggucca-----gguc
47551.1     401     446     NM_001010872.3  2688    2733    -       -2
GGCUC-UGGAGCGAGCCUGUGGAAAGGGGACACUUAGCCAAGG
:|    |:||:     |::||:|||:|:::|||  ||||  |||
ucacaaaucuuu----gguacuuuuucuuuugu--aucg--ucc
47551.1     553     599     NM_001010872.3  4747    4786    -       -2
CU---GCUGU--CGGAGAGAGAUGACGGGCAACGGCGUAUUCUCAG
     |||    |:||||:|:|::||:::     ||:      |||
uuagccgaacgcgucucuuuuguuguuua-gaacguc----uguc
47551.1     258     302     NM_001010872.3  1448    1492    -       -2
--UGCU-CUGGGAAAUGGGUGGGCAUUUGGGCUGGGGACCC-UGCC---CAC
  ::|: |||    |||::|||:||||||::   :|  ||   |||   |||
gaguggugacg---uacuuaccuguaaacuu---ucacgguaucgguuugug
47551.1     1838    1886    NM_001010872.3  59      108     -       -3
UCUGGAAUCAGGUCACCUUUACAUCUGCCU
|||:|||::||||           ||||:|||
agaucuuggucc-----------uagaugga
47551.1     78      113     NM_001010872.3  5973    5997    +       -2
```

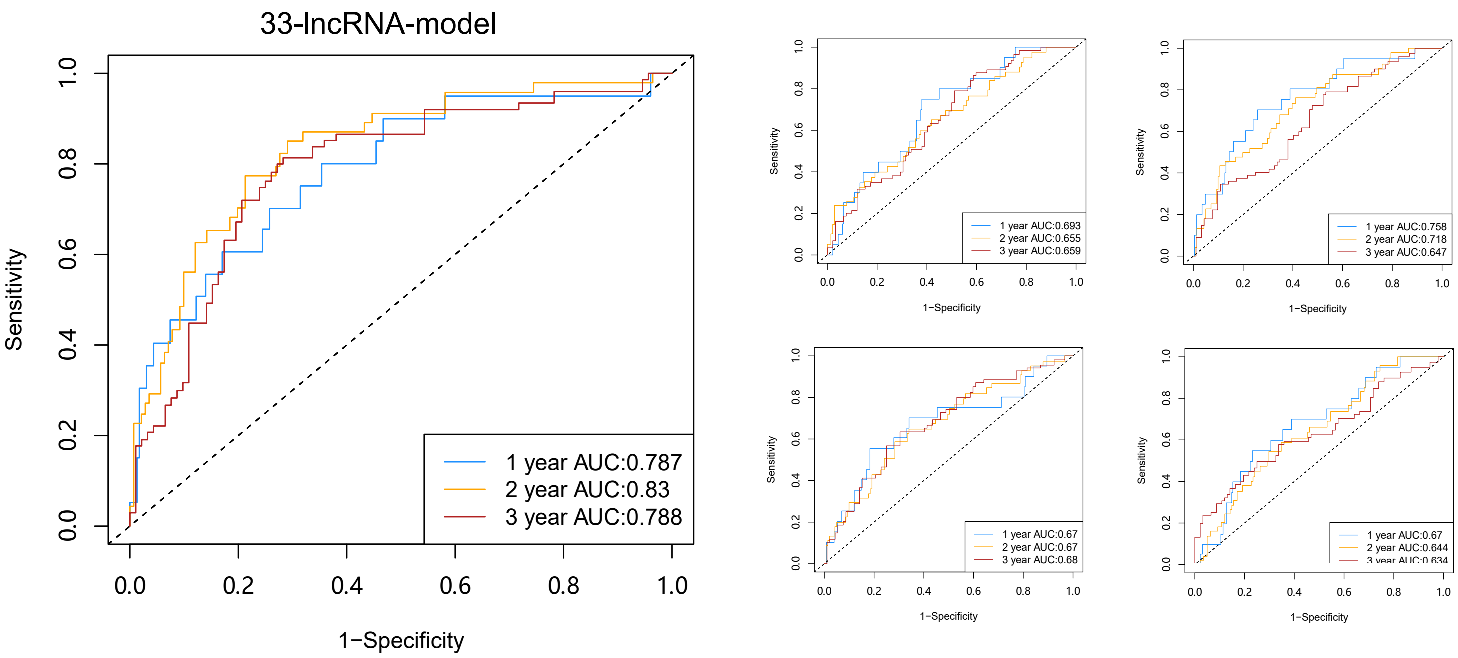

33-lncRNA-model
Sensitivity
1−Specificity
1 year AUC:0.787
2 year AUC:0.83
3 year AUC:0.788
1 year AUC:0.693
2 year AUC:0.655
3 year AUC:0.659
1 year AUC:0.758
2 year AUC:0.718
3 year AUC:0.647
1 year AUC:0.67
2 year AUC:0.67
3 year AUC:0.68
1 year AUC:0.67
2 year AUC:0.644
3 year AUC:0.634

Table S2. Clinical characteristics of patients with TCGA cervical cancer

Characteristic	CESC patients (n = 306)
**Age, year (mean ± SD)**	48.24±6.6
**Pathologic stage, n (%)**	
not reported	7(2.26)
I	163(52.75)
II	71(22.98)
III	46(14.89)
IV	22(7.12)
**T stage, n (%)**	
not reported	63(20.39)
T1	141(45.63)
T2	74(23.95)
T3	21(6.79)
T4	10(3.24)
**N stage, n (%)**	
not reported	111(35.92)
N0	136(44.01)
N1	62(20.07)
**M stage, n (%)**	
not reported	182(58.90)
M0	116(37.54)
M1	11(3.56)

**Deaths(%)**	23.95

Table S3. Primers and siRNAs used in this study

Primers used for qRT-PCR analyses

Gene name	sequences（5' to 3'）
GAPDH	F: ACCTGACCTGCCGTCTAGAA
	R: TCCACCACCCTGTTGCTGTA
FAM83B	F: TCCTCATCACGGGAAGGCTA
	R: TGTTTTTCTCTGCGCAAGCC
EGFR-AS1	F: AGTTATGCTGGGGAACAGCC
	R: TAGCCTTCCCGTGATGAGGA

siRNA sequences

Gene name	sequences（5' to 3'）
FAM83B siRNA(siFAM83B)	Sense: 5'-*GCAAGAAAGGUCAUUGCUUdTdT*-3'<br>Antisense: 5'- *AAGCAAUGACCUUUCUUGCdTdT*-3'
EGFR-AS1 siRNA(siEGFR-AS1)	Sense: 5'-*GAGGAGCACAUUGGAUAAAdTdT*-3'<br>Antisense: 5'- *UUUAUCCAAUGUGCUCCUCdTdT*-3'

Table S4. Antibodies used in the present study.

Protein name	Manufacture (cat. number)	Applications (working dilution)
GAPDH	Xianzhi Bio (AB-P-R 001)	WB (1:5000)
Akt	Bimake (A5031)	WB (1:1000)
p-Akt	Cell Signaling Technology (4060T)	WB (1:1000)
ERK	Cell Signaling Technology (4695S)	WB (1:2000)
p-ERK	Cell Signaling Technology (4370S)	WB (1:2000)

TableS5.Results of differential analysis of transcriptome data from 306 CESC patients

gene_name	baseMean	log2FoldChange	lfcSE	stat	pvalue	padj
TERT	6.130076	5.084918	1.180335	4.308029	1.65E-05	0.005806
DSG1	5.747417	4.990857	1.362296	3.663563	0.000249	0.028566
MAB21L3	5.725228	4.988455	1.183697	4.214299	2.51E-05	0.007317
H2AC13	5.564115	4.946846	1.180061	4.192025	2.76E-05	0.007696
OR2B6	5.416128	4.905535	1.180518	4.15541	3.25E-05	0.008402
TLX3	5.030033	4.798825	1.356826	3.536801	0.000405	0.037128
UGT1A6	4.995696	4.790665	1.285363	3.727091	0.000194	0.025062
TMPRSS11F	4.808506	4.7344	1.358703	3.484499	0.000493	0.040965
KRT31	4.781466	4.725843	1.361449	3.471186	0.000518	0.041896
EPGN	4.760632	4.720134	1.253646	3.765125	0.000166	0.023739
EVPLL	4.705943	4.706198	1.184231	3.974053	7.07E-05	0.013627
KRT16P1	4.640071	4.683045	1.23696	3.785932	0.000153	0.02285
POU4F1	4.469193	4.629553	1.320167	3.506793	0.000454	0.039454
B4GALNT2	4.389988	4.602838	1.353164	3.401536	0.00067	0.047057
LHX5	4.362129	4.592105	1.265092	3.629858	0.000284	0.030836
KRT3	4.319453	4.580172	1.230526	3.722125	0.000198	0.025241
CYP4F23P	4.138035	4.516721	1.248284	3.618344	0.000296	0.031874
GBX2	4.099309	4.502587	1.242647	3.623383	0.000291	0.031451
TPSP2	4.088269	4.502125	1.232738	3.652135	0.00026	0.028738
ATP12A	4.054873	4.487534	1.305037	3.438625	0.000585	0.044599
KRTAP4-1	3.934737	4.446118	1.241152	3.582251	0.000341	0.033448
CCR8	3.873654	4.431396	1.185377	3.738385	0.000185	0.024585
SLC34A1	3.885153	4.430931	1.198947	3.695687	0.000219	0.026719
KRT74	3.86626	4.421851	1.200377	3.683719	0.00023	0.027288

H4C4	3.803528	4.398447	1.181176	3.723788	0.000196	0.025233
GAST	3.792302	4.39061	1.27432	3.445454	0.00057	0.043709
CBY2	3.748411	4.374787	1.261352	3.468332	0.000524	0.041984
RNF225	3.701899	4.360452	1.224958	3.559675	0.000371	0.035617
H2AC14	3.633124	4.332351	1.183217	3.6615	0.000251	0.028636
HAPLN1	6.992248	4.311539	1.180354	3.652751	0.000259	0.028738
H2AC16	3.328456	4.205937	1.182798	3.555921	0.000377	0.035617
H3C12	3.290407	4.189156	1.182607	3.542306	0.000397	0.036691
TTC6	6.405166	4.187398	1.180563	3.546949	0.00039	0.036214
FAM172BP	3.24068	4.170087	1.21384	3.435452	0.000592	0.044817
MCIDAS	6.323285	4.16923	1.179852	3.53369	0.00041	0.0374
DLL3	6.148234	4.125442	1.180747	3.493924	0.000476	0.040043
LRRC37A9P	3.105031	4.113903	1.193651	3.446487	0.000568	0.043709
APOC2	3.037195	4.073354	1.187314	3.430728	0.000602	0.045165
MAJIN	5.87544	4.061175	1.186591	3.422558	0.00062	0.045292
PGAM1P7	2.898842	4.012389	1.18407	3.388641	0.000702	0.048151
ZIC5	7.856393	3.000495	0.836545	3.586772	0.000335	0.033064
PLA2G4E	7.720381	2.969276	0.868528	3.418747	0.000629	0.045425
IGF2BP3	7.668043	2.966888	0.841867	3.524178	0.000425	0.038256
H3C8	7.142535	2.861645	0.83548	3.425151	0.000614	0.045194
C5orf66-AS1	7.61182	2.535571	0.726206	3.491533	0.00048	0.040237
OTX1	9.289717	2.503197	0.64709	3.86839	0.00011	0.018217
ZIC2	8.888024	2.440562	0.648684	3.76233	0.000168	0.023739
PADI3	8.040368	2.2995	0.676185	3.400696	0.000672	0.047057
LHX2	7.742706	2.24175	0.648522	3.456706	0.000547	0.043161
FOXA1	10.75252	2.227989	0.547468	4.069625	4.71E-05	0.010889
FOXN1	9.210165	2.225812	0.595868	3.735414	0.000187	0.024591
HOXC13	7.648936	2.219578	0.655145	3.387917	0.000704	0.048151
KRT78	7.618565	2.21459	0.654138	3.385507	0.00071	0.048253
KLRG2	7.605012	2.21414	0.649535	3.408808	0.000652	0.046314
DQX1	9.117934	2.212237	0.591301	3.741306	0.000183	0.024502
NMU	9.796134	2.092134	0.547674	3.820039	0.000133	0.020822
LGALS7B	9.784242	2.087745	0.577881	3.612762	0.000303	0.032098
ZYG11A	8.259621	2.070764	0.591935	3.498294	0.000468	0.039906
CALML5	10.19575	1.951577	0.56995	3.424117	0.000617	0.045194
PLA2G4F	8.642853	1.911587	0.548929	3.482391	0.000497	0.040965
SYT8	9.513144	1.855724	0.514933	3.603818	0.000314	0.032164
NEIL3	8.740412	1.736057	0.512646	3.386466	0.000708	0.048245
FAM83B	9.588259	1.699323	0.483815	3.51234	0.000444	0.039309
FOXE1	9.343314	1.661493	0.492997	3.370192	0.000751	0.049061
KLC3	9.174789	1.633387	0.484103	3.37405	0.000741	0.049004
GSDMC	10.09023	1.61937	0.459803	3.521881	0.000428	0.038287
CKMT1B	9.912149	1.591635	0.458831	3.46889	0.000523	0.041984

E2F7	10.59918	1.55481	0.437409	3.554587	0.000379	0.035617
OVOL1	10.44745	1.530701	0.437584	3.49807	0.000469	0.039906
CRYBG2	11.35776	1.525857	0.419028	3.64142	0.000271	0.029641
GPR87	10.99125	1.480086	0.422362	3.504304	0.000458	0.039656
ANXA8	10.80972	1.454811	0.424293	3.428792	0.000606	0.045194
E2F2	10.98646	1.362024	0.402613	3.382963	0.000717	0.048381
PITX1	13.41292	1.349985	0.363047	3.718489	0.0002	0.025448
IQANK1	11.51124	1.322999	0.388033	3.4095	0.000651	0.046314
DLGAP5	11.40455	1.309587	0.38799	3.375316	0.000737	0.049004
ASPM	11.90738	1.272481	0.374893	3.394255	0.000688	0.04757
UHRF1	11.81723	1.261869	0.374913	3.365762	0.000763	0.049226
NAP1L3	5.13053	-1.00419	0.272249	-3.68848	0.000226	0.027129
FGF7	6.455067	-1.00507	0.24704	-4.06844	4.73E-05	0.010889
ACTG2	8.63044	-1.00615	0.208875	-4.81697	1.46E-06	0.001192
PIANP	5.458398	-1.00886	0.263644	-3.82661	0.00013	0.020589
BHMT2	4.96097	-1.00918	0.289727	-3.48321	0.000495	0.040965
HTR2B	5.575897	-1.01947	0.260433	-3.91451	9.06E-05	0.015964
NCAM1	6.870682	-1.02726	0.231638	-4.43477	9.22E-06	0.003624
HRC	4.404019	-1.02983	0.289788	-3.55372	0.00038	0.035617
PCDHGA2	4.06312	-1.03112	0.303208	-3.40071	0.000672	0.047057
F10	5.713336	-1.03131	0.254912	-4.04576	5.22E-05	0.011323
SEC14L6	4.556412	-1.03246	0.29934	-3.44912	0.000562	0.043709
NECAB1	5.341577	-1.03865	0.265964	-3.90523	9.41E-05	0.016309
CNN1	8.594944	-1.03964	0.206485	-5.03492	4.78E-07	0.000698
TCEAL7	5.444442	-1.05554	0.258417	-4.08466	4.41E-05	0.010743
FREM1	5.5676	-1.06614	0.275612	-3.86828	0.00011	0.018217
EXTL1	3.650034	-1.07209	0.318326	-3.3679	0.000757	0.049156
PGR	6.924796	-1.08025	0.237482	-4.54878	5.40E-06	0.00269
PRTG	4.088531	-1.0833	0.308754	-3.50862	0.00045	0.039454
ZNF582-AS1	3.904205	-1.09025	0.305243	-3.57174	0.000355	0.034521
NRXN3	4.684418	-1.09159	0.30282	-3.60475	0.000312	0.032164
DIRAS1	5.479143	-1.09367	0.26352	-4.15025	3.32E-05	0.008487
MGARP	3.739129	-1.09404	0.30972	-3.53233	0.000412	0.037425
MYRIP	4.969975	-1.10158	0.286763	-3.84143	0.000122	0.019689
LRRC2	4.776555	-1.11259	0.277959	-4.00271	6.26E-05	0.012429
DES	8.480856	-1.11476	0.206844	-5.38935	7.07E-08	0.000161
PTGIS	6.582658	-1.12164	0.232845	-4.81709	1.46E-06	0.001192
PCDHB5	4.436679	-1.12223	0.285278	-3.9338	8.36E-05	0.015085
CYTL1	4.586654	-1.12588	0.279163	-4.03305	5.51E-05	0.011485
HSPB7	6.088285	-1.13158	0.238757	-4.73946	2.14E-06	0.001511
TMEM108	3.795778	-1.13272	0.328551	-3.44762	0.000566	0.043709
NGF	4.091699	-1.13758	0.330202	-3.44509	0.000571	0.043709
BMPER	4.243937	-1.13761	0.332617	-3.42018	0.000626	0.045425
NOVA1	4.679414	-1.14201	0.280754	-4.06766	4.75E-05	0.010889

ALKAL2	4.358773	-1.14783	0.307679	-3.73063	0.000191	0.02487
GSTM5	5.714559	-1.14829	0.24764	-4.63694	3.54E-06	0.001954
MAMDC2	5.852101	-1.15068	0.250396	-4.59543	4.32E-06	0.002264
PCDHB6	3.601346	-1.15091	0.338611	-3.39891	0.000677	0.047203
THPO	3.453341	-1.15123	0.333422	-3.45278	0.000555	0.043625
C8orf88	4.039515	-1.15226	0.334087	-3.44897	0.000563	0.043709
STAC2	4.339118	-1.15983	0.327555	-3.54087	0.000399	0.036724
TACR1	4.926418	-1.16002	0.270496	-4.28847	1.80E-05	0.005959
HSPB2	4.179774	-1.16041	0.286786	-4.04628	5.20E-05	0.011323
CSDC2	5.817943	-1.1605	0.244623	-4.74402	2.10E-06	0.001511
CADM3	4.464302	-1.16563	0.317262	-3.67404	0.000239	0.027731
ZNF835	3.707679	-1.16706	0.305972	-3.81429	0.000137	0.020923
TRPC4	4.887959	-1.16926	0.263253	-4.4416	8.93E-06	0.003579
PKNOX2	4.291424	-1.17229	0.310837	-3.77139	0.000162	0.023706
ANGPT4	3.395626	-1.17471	0.326361	-3.59941	0.000319	0.032345
HPSE2	4.720921	-1.17793	0.320856	-3.67123	0.000241	0.02788
ADAMTS8	4.42645	-1.17871	0.282996	-4.16509	3.11E-05	0.008157
LINC01197	4.114617	-1.18212	0.286316	-4.12873	3.65E-05	0.009094
FAM180A	3.956754	-1.18461	0.299513	-3.95513	7.65E-05	0.014346
GFRA1	5.275802	-1.18856	0.26549	-4.47687	7.57E-06	0.003295
HSD17B13	3.628323	-1.19917	0.320048	-3.74683	0.000179	0.024502
HIPK4	2.899084	-1.20165	0.341417	-3.5196	0.000432	0.038414
ADCYAP1R1	4.196049	-1.20674	0.322581	-3.74088	0.000183	0.024502
KCNMB2	3.462301	-1.20905	0.323701	-3.73508	0.000188	0.024591
ADRA1D	3.606079	-1.20937	0.313809	-3.85383	0.000116	0.018867
JPH4	4.895094	-1.21243	0.25992	-4.66464	3.09E-06	0.001846
ABCA8	5.004089	-1.22228	0.264905	-4.61403	3.95E-06	0.002125
HAND2	5.574965	-1.22233	0.24605	-4.9678	6.77E-07	0.000706
PTGER3	5.544931	-1.23026	0.247758	-4.96558	6.85E-07	0.000706
GPR182	2.543368	-1.23321	0.366078	-3.36872	0.000755	0.049156
ACR	2.553483	-1.23409	0.366165	-3.3703	0.000751	0.049061
CCDC89	3.115761	-1.23454	0.335376	-3.68107	0.000232	0.027288
MAGEL2	3.533966	-1.23624	0.342724	-3.6071	0.00031	0.032164
PEG3	5.058108	-1.24748	0.255014	-4.89182	9.99E-07	0.000973
STUM	4.076577	-1.24802	0.34149	-3.65462	0.000258	0.028738
RBM24	4.336407	-1.2509	0.291999	-4.2839	1.84E-05	0.005959
GREM2	4.198086	-1.25103	0.297197	-4.20943	2.56E-05	0.007371
ACTN2	2.941951	-1.25242	0.366869	-3.41382	0.000641	0.045939
ADGRD1	4.758129	-1.2542	0.268353	-4.67368	2.96E-06	0.001846
SLC35F1	3.353842	-1.25427	0.318628	-3.93646	8.27E-05	0.015085
PGM5	6.448481	-1.25461	0.227373	-5.51783	3.43E-08	0.000161
C1QTNF7	4.60847	-1.25506	0.279197	-4.49524	6.95E-06	0.003089
SLC1A7	3.873101	-1.27266	0.320474	-3.97119	7.15E-05	0.013655
PTGFR	4.55018	-1.27546	0.285578	-4.46622	7.96E-06	0.003391

NPY1R	4.130769	-1.2771	0.356608	-3.58125	0.000342	0.033448
CCL23	3.43716	-1.28009	0.31884	-4.01485	5.95E-05	0.01204
CHRDL2	5.349648	-1.28455	0.256011	-5.01757	5.23E-07	0.000706
PABPC5	3.263612	-1.29102	0.317966	-4.06024	4.90E-05	0.011013
SGCA	4.232874	-1.29124	0.277064	-4.66044	3.16E-06	0.001846
NUDT10	4.067389	-1.29811	0.311509	-4.16715	3.08E-05	0.008157
ASPA	4.062094	-1.30042	0.304755	-4.2671	1.98E-05	0.006228
FHL5	3.918703	-1.30384	0.288321	-4.52218	6.12E-06	0.002895
DUSP26	3.109378	-1.30816	0.347705	-3.76227	0.000168	0.023739
FXYD1	4.323684	-1.3084	0.26993	-4.84717	1.25E-06	0.001113
LPAR4	2.421984	-1.30864	0.375449	-3.48553	0.000491	0.040965
ACTC1	4.047769	-1.31125	0.316997	-4.13649	3.53E-05	0.0089
GDF10	3.344846	-1.3191	0.366532	-3.59887	0.00032	0.032345
NAP1L2	3.877836	-1.31932	0.293495	-4.4952	6.95E-06	0.003089
LONRF2	5.215268	-1.3208	0.374558	-3.5263	0.000421	0.038119
C1orf229	2.676891	-1.32127	0.362716	-3.64272	0.00027	0.029641
KCNA5	3.046594	-1.3341	0.330122	-4.04122	5.32E-05	0.011323
C16orf89	3.997328	-1.33427	0.389623	-3.42452	0.000616	0.045194
RSPO1	4.482161	-1.33861	0.316889	-4.22421	2.40E-05	0.007207
NTRK3	3.963634	-1.33862	0.301212	-4.44411	8.83E-06	0.003579
WT1	5.115253	-1.34742	0.289526	-4.65389	3.26E-06	0.00185
PLN	5.37628	-1.34824	0.248497	-5.42558	5.78E-08	0.000161
CABCOCO1	3.120348	-1.36207	0.401427	-3.39308	0.000691	0.04757
KLHL10	1.944222	-1.364	0.401601	-3.3964	0.000683	0.047476
CDO1	4.007192	-1.36939	0.302351	-4.52914	5.92E-06	0.002883
SLC5A4	2.446411	-1.37482	0.365325	-3.76329	0.000168	0.023739
PURG	2.808391	-1.38465	0.345176	-4.01141	6.04E-05	0.012097
CCDC85A	2.919044	-1.38615	0.402048	-3.44773	0.000565	0.043709
OMD	3.942122	-1.39182	0.360985	-3.85562	0.000115	0.018867
C20orf203	2.156732	-1.39707	0.38776	-3.60292	0.000315	0.032164
WSCD2	4.06611	-1.3975	0.39839	-3.50787	0.000452	0.039454
ATP1A2	3.936846	-1.39811	0.327586	-4.26793	1.97E-05	0.006228
PLK5	2.915595	-1.40643	0.389355	-3.61221	0.000304	0.032098
NKAPL	2.510801	-1.41138	0.349806	-4.03475	5.47E-05	0.011485
RBFOX3	3.649416	-1.41564	0.284911	-4.96873	6.74E-07	0.000706
MYOCD	4.480496	-1.42676	0.266433	-5.35505	8.55E-08	0.000175
VEGFD	3.939034	-1.44299	0.275814	-5.23176	1.68E-07	0.000264
BCHE	4.17984	-1.44467	0.370791	-3.89617	9.77E-05	0.016788
ART4	2.317589	-1.45334	0.428273	-3.39348	0.00069	0.04757
PCDHGA3	2.303945	-1.45648	0.39855	-3.65445	0.000258	0.028738
ZBTB16	3.633534	-1.46853	0.311869	-4.70881	2.49E-06	0.001698
TPSG1	2.427109	-1.471	0.43034	-3.41823	0.00063	0.045425
ANGPTL1	4.328568	-1.4769	0.273941	-5.39131	6.99E-08	0.000161
PTCHD1	3.611879	-1.47861	0.422362	-3.50081	0.000464	0.039842

GPIHBP1	3.656123	-1.50618	0.284057	-5.30241	1.14E-07	0.000212
AGTR1	2.935267	-1.51161	0.413785	-3.65312	0.000259	0.028738
AQP8	1.764077	-1.513	0.432117	-3.50137	0.000463	0.039842
PI16	4.340549	-1.51929	0.320528	-4.73996	2.14E-06	0.001511
RASA4B	1.973114	-1.52573	0.39045	-3.90762	9.32E-05	0.016286
C7	4.891672	-1.52814	0.398794	-3.8319	0.000127	0.020308
GALNT17	3.47909	-1.53359	0.361917	-4.2374	2.26E-05	0.0069
SCRG1	3.0063	-1.5346	0.316513	-4.84846	1.24E-06	0.001113
DNM1P46	2.073193	-1.53588	0.419735	-3.65915	0.000253	0.028738
TRPM3	2.306905	-1.54037	0.429396	-3.58729	0.000334	0.033064
MYL3	2.648369	-1.54305	0.35142	-4.3909	1.13E-05	0.004271
HFM1	2.752038	-1.5444	0.387413	-3.98644	6.71E-05	0.013106
ATRNL1	3.658026	-1.54993	0.417708	-3.71055	0.000207	0.025794
RCVRN	1.942362	-1.55042	0.451321	-3.43529	0.000592	0.044817
RGS22	2.947633	-1.56542	0.425592	-3.67821	0.000235	0.027437
ERVFRD-1	1.65836	-1.59423	0.443833	-3.59196	0.000328	0.032729
TCEAL2	3.885142	-1.59564	0.465698	-3.42635	0.000612	0.045194
OGN	4.714935	-1.61107	0.293777	-5.48398	4.16E-08	0.000161
GPR22	2.190893	-1.61245	0.382009	-4.22098	2.43E-05	0.007207
WDR17	2.407085	-1.61474	0.397187	-4.06545	4.79E-05	0.010889
ZCCHC12	3.897488	-1.6335	0.299744	-5.44965	5.05E-08	0.000161
BPI	2.496298	-1.63477	0.435085	-3.75736	0.000172	0.024043
ZNF208	2.485616	-1.63648	0.348674	-4.69344	2.69E-06	0.001772
ECRG4	3.311323	-1.65511	0.449131	-3.68514	0.000229	0.027288
CASQ2	3.492739	-1.66693	0.318579	-5.2324	1.67E-07	0.000264
PTTG2	1.478553	-1.6691	0.439491	-3.79781	0.000146	0.022106
MEOX2	2.852857	-1.66923	0.393193	-4.24532	2.18E-05	0.006761
CRHBP	2.851469	-1.72108	0.302158	-5.69596	1.23E-08	0.000161
CCL14	3.366888	-1.72149	0.436167	-3.94685	7.92E-05	0.014584
CHRDL1	4.27675	-1.72542	0.497094	-3.47101	0.000519	0.041896
CA4	2.635283	-1.73531	0.471303	-3.68193	0.000231	0.027288
NPAS4	2.215863	-1.73834	0.442746	-3.92628	8.63E-05	0.015336
TCF21	3.129499	-1.7394	0.468374	-3.7137	0.000204	0.025774
KLHL4	2.672891	-1.75741	0.436392	-4.02715	5.65E-05	0.011658
KIAA1210	1.799782	-1.75917	0.515443	-3.41292	0.000643	0.045939
DPP6	3.758155	-1.76762	0.355547	-4.97155	6.64E-07	0.000706
SCN2B	2.567386	-1.76891	0.449836	-3.93235	8.41E-05	0.015085
TRIM63	2.244401	-1.85307	0.476265	-3.89084	9.99E-05	0.016878
KCNB1	2.881103	-1.86045	0.399227	-4.66013	3.16E-06	0.001846
GDAP1L1	1.648403	-1.88078	0.421678	-4.46023	8.19E-06	0.003416
HNRNPA1P15	0.980856	-1.91197	0.517396	-3.69536	0.00022	0.026719
CASP12	2.025955	-1.93496	0.350784	-5.5161	3.47E-08	0.000161
ANGPTL7	1.76495	-1.94015	0.536368	-3.61721	0.000298	0.031874

CTNNA3	2.107067	-1.94295	0.479758	-4.04985	5.13E-05	0.011323
GPM6A	2.972583	-1.95171	0.410606	-4.75324	2.00E-06	0.001511
SCN7A	2.669686	-1.97495	0.495525	-3.98558	6.73E-05	0.013106
RPL21P135	1.274836	-1.98924	0.455326	-4.36882	1.25E-05	0.00456
PRND	2.281756	-1.99782	0.490574	-4.07241	4.65E-05	0.010889
CD300LG	2.204106	-2.05294	0.477773	-4.29688	1.73E-05	0.005959
CMTM5	1.615677	-2.07011	0.492862	-4.20019	2.67E-05	0.007572
DGKB	2.203223	-2.10734	0.600344	-3.51023	0.000448	0.039452
FAM180B	1.335472	-2.11247	0.461226	-4.58011	4.65E-06	0.002375
WNT9B	1.45388	-2.14997	0.62759	-3.42575	0.000613	0.045194
MYOC	1.965233	-2.16835	0.626259	-3.46238	0.000535	0.042756
ZNF99	1.11681	-2.18105	0.5205	-4.1903	2.79E-05	0.007696
CCL16	1.409877	-2.1977	0.500953	-4.38703	1.15E-05	0.004271
OPCML	1.771185	-2.20361	0.526871	-4.18245	2.88E-05	0.007861
MYOZ2	1.472053	-2.20368	0.548019	-4.02117	5.79E-05	0.011839
SYT9	1.541975	-2.20988	0.611926	-3.61135	0.000305	0.032098
ADH1B	2.79716	-2.24797	0.647013	-3.47438	0.000512	0.041703
C1QTNF9	1.358568	-2.2512	0.593986	-3.78999	0.000151	0.022645
KLHL33	1.152495	-2.31839	0.541012	-4.28527	1.83E-05	0.005959
CMA1	1.699371	-2.3283	0.615787	-3.78102	0.000156	0.023137
ZNF676	1.307785	-2.38563	0.645865	-3.6937	0.000221	0.026735
LCN6	1.835611	-2.38714	0.553502	-4.31279	1.61E-05	0.005782
LCN10	1.551255	-2.40527	0.69214	-3.47512	0.000511	0.041703
TCF23	2.283826	-2.51428	0.556453	-4.5184	6.23E-06	0.002895
MAS1	1.295056	-2.54646	0.512987	-4.96399	6.91E-07	0.000706
SEL1L2	1.068007	-2.77376	0.512327	-5.41403	6.16E-08	0.000161

TableS6.co-expression network of mRNA and lncRNA

pearson_coef	p_value	mRNA	lncRNA
0.92	0	KRT16P1	KRT16P3
0.92	0	HOXC13	HOXC13-AS
0.86	0	DSG1	DSG1-AS1
0.82	0	CCL14	CADM3-AS1
0.78	0	PLA2G4E	PLA2G4E-AS
0.78	0	OVOL1	OVOL1-AS1
0.76	0	DSG1	LINC01527
0.76	0	TMPRSS11F	DSG1-AS1
0.75	0	OGN	HAND2-AS1
0.74	0	FOXN1	LINC00640
0.74	0	TCEAL2	HAND2-AS1
0.73	0	SLC34A1	LINC00640
0.72	0	LCN6	CADM3-AS1
0.72	0	TCF21	ACTA2-AS1
0.71	0	FOXN1	CALML3-AS1

0.7	0	TMPRSS11F	LINC01527
0.7	0	FOXN1	CERS3-AS1
0.69	0	GSDMC	MIR205HG
0.69	0	AGTR1	MAGI2-AS3
0.69	0	KRT31	LINC00640
0.69	0	DSG1	KRT16P3
0.69	0	GSDMC	IL20RB-AS1
0.69	0	TMPRSS11F	IL20RB-AS1
0.69	0	C7	CADM3-AS1
0.68	0	TMPRSS11F	SH3PXD2A-A
0.68	0	SLC34A1	LINC02541
0.68	0	CCR8	LINC02099
0.68	0	FOXN1	KRT16P3
0.68	0	CCL14	HAND2-AS1
0.68	0	GSDMC	FAM83A-AS1
0.68	0	AGTR1	DNM3OS
0.68	0	TMPRSS11F	CALML3-AS1
0.67	0	CRYBG2	SH3PXD2A-A
0.67	0	TMPRSS11F	MLIP-IT1
0.67	0	FOXN1	MIR205HG
0.67	0	MYL3	MEG3
0.67	0	RNF225	LINC02560
0.67	0	KRT78	LINC01527
0.67	0	KRT16P1	DSG1-AS1
0.67	0	TCF21	CARMN
0.67	0	LCN10	CADM3-AS1
0.67	0	FOXN1	AATBC
0.66	0	TMPRSS11F	SMILR
0.66	0	TCEAL2	MAGI2-AS3
0.66	0	TMPRSS11F	KRT16P3
0.66	0	FOXN1	IL20RB-AS1
0.66	0	DPP6	HAND2-AS1
0.66	0	KRT78	DSG1-AS1
0.66	0	OGN	CARMN
0.66	0	OGN	CADM3-AS1
0.66	0	SLC34A1	AATBC
0.65	0	DSG1	SMILR
0.65	0	OGN	MIR497HG
0.65	0	MYL3	MAGI2-AS3
0.65	0	CRYBG2	LINC02560
0.65	0	APOC2	LINC01857
0.65	0	KRT74	LINC00640
0.65	0	KRT3	DSG1-AS1
0.65	0	KRT74	CALML3-AS1

0.65	0	SLC34A1	CALML3-AS1
0.65	0	TCEAL2	ADAMTS9-AS2
0.65	0	OGN	ADAMTS9-AS1
0.64	0	CRYBG2	OVOL1-AS1
0.64	0	DSG1	MLIP-IT1
0.64	0	SLC34A1	MIR205HG
0.64	0	CHRDL1	MAGI2-AS3
0.64	0	FAM83B	LINC02541
0.64	0	GSDMC	LINC02541
0.64	0	KRT16P1	LINC01527
0.64	0	KRT3	LINC01527
0.64	0	LGALS7B	LINC00640
0.64	0	TMPRSS11F	LINC00640
0.64	0	APOC2	L3MBTL4-AS
0.64	0	PLA2G4E	KRT16P3
0.64	0	CRYBG2	IL20RB-AS1
0.64	0	FOXN1	DSG1-AS1
0.64	0	TMPRSS11F	CERS3-AS1
0.64	0	SCN7A	ADAMTS9-AS2
0.64	0	ZNF208	ADAMTS9-AS2
0.63	0	DSG1	SH3PXD2A-A
0.63	0	KRT78	SH3PXD2A-A
0.63	0	TMPRSS11F	OVOL1-AS1
0.63	0	GSDMC	MYOSLID
0.63	0	FOXN1	MLIP-IT1
0.63	0	SLC34A1	MIR3659HG
0.63	0	LRRC37A9P	MAP3K14-AS
0.63	0	OGN	MAGI2-AS3
0.63	0	CRYBG2	LINC02178
0.63	0	ECRG4	LINC01936
0.63	0	KRT16P1	LINC01605
0.63	0	DSG1	LINC00640
0.63	0	KLRG2	LINC00640
0.63	0	PLA2G4E	IL20RB-AS1
0.63	0	C7	HAND2-AS1
0.63	0	ZCCHC12	HAND2-AS1
0.63	0	MYL3	DNM3OS
0.63	0	TMPRSS11F	CLCA3P
0.63	0	SLC34A1	CERS3-AS1
0.63	0	MYL3	CARMN
0.63	0	GPIHBP1	CADM3-AS1
0.63	0	CCL14	ADAMTS9-AS2
0.63	0	C7	ADAMTS9-AS1
0.63	0	SCN7A	ADAMTS9-AS1

0.63	0	MYL3	ACTA2-AS1
0.62	0	CRYBG2	SMILR
0.62	0	KRT78	SMILR
0.62	0	OVOL1	SH3PXD2A-A
0.62	0	APOC2	PCED1B-AS1
0.62	0	OGN	MRGPRF-AS1
0.62	0	KLC3	MIR205HG
0.62	0	KRT16P1	MIR205HG
0.62	0	TCF21	MAGI2-AS3
0.62	0	FOXN1	LINC02560
0.62	0	FOXN1	LINC01679
0.62	0	CRYBG2	LINC01527
0.62	0	FOXN1	LINC01527
0.62	0	TMPRSS11F	LINC00519
0.62	0	GSDMC	KRT16P3
0.62	0	BPI	HAND2-AS1
0.62	0	CHRDL1	HAND2-AS1
0.62	0	MYL3	HAND2-AS1
0.62	0	CRYBG2	DSG1-AS1
0.62	0	KRT31	DSG1-AS1
0.62	0	TCF21	DNM3OS
0.62	0	AGTR1	CARMN
0.62	0	SCN7A	CARMN
0.62	0	CRYBG2	CALML3-AS1
0.62	0	GSDMC	CALML3-AS1
0.62	0	KRT31	CALML3-AS1
0.62	0	GSDMC	C10orf55
0.62	0	CHRDL1	ADAMTS9-AS1
0.62	0	GPIHBP1	ADAMTS9-AS1
0.62	0	TCEAL2	ADAMTS9-AS1
0.62	0	OGN	ACTA2-AS1
0.61	0	MYL3	PTPRD-AS1
0.61	0	LRRC37A9P	PAXIP1-AS2
0.61	0	FOXN1	OVOL1-AS1
0.61	0	TCEAL2	MRGPRF-AS1
0.61	0	CRYBG2	MIR205HG
0.61	0	KRT74	MIR205HG
0.61	0	KRT78	LINC02560
0.61	0	FOXN1	LINC02541
0.61	0	CCR8	LINC02325
0.61	0	TMPRSS11F	LINC02031
0.61	0	CCR8	LINC00861
0.61	0	KRT16P1	LINC00640
0.61	0	KRT16P1	LINC00520

0.61	0	SLC34A1	LINC00519
0.61	0	FAM83B	IL20RB-AS1
0.61	0	KRT16P1	IL20RB-AS1
0.61	0	GPR22	HAND2-AS1
0.61	0	LCN6	HAND2-AS1
0.61	0	E2F7	EP300-AS1
0.61	0	CCR8	EML4-AS1
0.61	0	OVOL1	DSG1-AS1
0.61	0	DSG1	CERS3-AS1
0.61	0	C7	CARMN
0.61	0	GPIHBP1	CARMN
0.61	0	ZNF208	CARMN
0.61	0	DSG1	CALML3-AS1
0.61	0	APOC2	APOC1P1
0.61	0	OGN	ADAMTS9-AS2
0.61	0	DPP6	ADAMTS9-AS1
0.61	0	TCF23	ADAMTS9-AS1
0.61	0	TCEAL2	ACTA2-AS1
0.61	0	KLRG2	AATBC
0.6	0	E2F7	YEATS2-AS1
0.6	0	TCEAL2	WT1-AS
0.6	0	PLA2G4E	SH3PXD2A-A
0.6	0	UGT1A6	NMRAL2P
0.6	0	DPP6	MRGPRF-AS1
0.6	0	LCN6	MRGPRF-AS1
0.6	0	MYL3	MRGPRF-AS1
0.6	0	CRYBG2	MLIP-IT1
0.6	0	GPIHBP1	MIR497HG
0.6	0	FOXN1	MIR3659HG
0.6	0	CCL14	MEF2C-AS1
0.6	0	ZNF208	MAGI2-AS3
0.6	0	CRYBG2	LINC02595
0.6	0	TMPRSS11F	LINC02595
0.6	0	TMPRSS11F	LINC02541
0.6	0	GPIHBP1	LINC02202
0.6	0	TCEAL2	LINC01936
0.6	0	TCF21	LINC01614
0.6	0	DSG1	LINC01605
0.6	0	KRT3	LINC01395
0.6	0	DSG1	LINC00520
0.6	0	PLA2G4E	LINC00520
0.6	0	FOXN1	LINC00519
0.6	0	KRT74	LINC00519
0.6	0	CRYBG2	KRT16P3

0.6	0	KRT78	KRT16P3
0.6	0	DSG1	IL20RB-AS1
0.6	0	OVOL1	IL20RB-AS1
0.6	0	SCN7A	HAND2-AS1
0.6	0	RNF225	GRHL3-AS1
0.6	0	TMPRSS11F	GRHL3-AS1
0.6	0	CRYBG2	FAM83A-AS1
0.6	0	BPI	EMX2OS
0.6	0	C5rf66-AS1	DSG1-AS1
0.6	0	TCEAL2	CARMN
0.6	0	BPI	ADAMTS9-AS2
0.6	0	C7	ADAMTS9-AS2
0.6	0	ZNF676	ADAMTS9-AS2
0.6	0	CCL14	ADAMTS9-AS1
0.6	0	ECRG4	ADAMTS9-AS1
0.6	0	MYL3	ADAMTS9-AS1
0.6	0	GPIHBP1	ACTA2-AS1
0.6	0	SCRG1	ACTA2-AS1
0.59	0	CCR8	TRBV11-2
0.59	0	GSDMC	SMILR
0.59	0	FOXN1	SH3PXD2A-A
0.59	0	GSDMC	SH3PXD2A-A
0.59	0	GPIHBP1	RGS5
0.59	0	TCF21	PTPRD-AS1
0.59	0	DSG1	OVOL1-AS1
0.59	0	FAM83B	OVOL1-AS1
0.59	0	KRT78	MLIP-IT1
0.59	0	SLC34A1	MLIP-IT1
0.59	0	MAJIN	MIR9-3HG
0.59	0	FOXN1	MIR4713HG
0.59	0	KLRG2	MIR205HG
0.59	0	CASP12	MAGI2-AS3
0.59	0	OVOL1	LINC02595
0.59	0	APOC2	LINC01943
0.59	0	MYL3	LINC01936
0.59	0	CRYBG2	LINC01605
0.59	0	KRT31	LINC01527
0.59	0	PLA2G4E	LINC01527
0.59	0	CCR8	LINC00426
0.59	0	SLC34A1	KRT16P3
0.59	0	SLC34A1	IL20RB-AS1
0.59	0	FAM83B	GRHL3-AS1
0.59	0	TCEAL2	EMX2OS
0.59	0	OGN	DNM3OS

0.59	0	DSG1	CT69
0.59	0	GSDMC	CERS3-AS1
0.59	0	KRT16P1	CERS3-AS1
0.59	0	FAM83B	CD44-AS1
0.59	0	CASP12	CARMN
0.59	0	CHRDL1	CARMN
0.59	0	KRT16P1	CALML3-AS1
0.59	0	KRT78	CALML3-AS1
0.59	0	OVOL1	CALML3-AS1
0.59	0	PI16	CADM3-AS1
0.59	0	FOXN1	C5rf34-AS1
0.59	0	CRYBG2	C10orf55
0.59	0	LCN6	ADAMTS9-AS1
0.59	0	AGTR1	ACTA2-AS1
0.59	0	SCN7A	ACTA2-AS1
0.58	0	SLC34A1	STEAP3-AS1
0.58	0	TMPRSS11F	PLA2G4E-AS
0.58	0	ZIC2	PCCA-DT
0.58	0	GSDMC	MLIP-IT1
0.58	0	LGALS7B	MIR205HG
0.58	0	C7	MEF2C-AS1
0.58	0	OGN	MEF2C-AS1
0.58	0	C7	MAGI2-AS3
0.58	0	MEOX2	MAGI2-AS3
0.58	0	SCN7A	MAGI2-AS3
0.58	0	APOC2	LINC02528
0.58	0	CASP12	LINC02202
0.58	0	KRT78	LINC02031
0.58	0	SLC34A1	LINC02031
0.58	0	GSDMC	LINC01679
0.58	0	GSDMC	LINC01527
0.58	0	OVOL1	LINC01527
0.58	0	FOXN1	LINC00964
0.58	0	KRT3	LINC00964
0.58	0	TMPRSS11F	LINC00520
0.58	0	KRT31	LINC00519
0.58	0	FOXE1	IL20RB-AS1
0.58	0	MYL3	IGF2-AS
0.58	0	CASP12	HAND2-AS1
0.58	0	GPIHBP1	HAND2-AS1
0.58	0	RGS22	HAND2-AS1
0.58	0	ZNF208	HAND2-AS1
0.58	0	KRTAP4-1	FMO9P
0.58	0	CCL14	EMX2OS

0.58	0	LRRC37A9P	DLEU2L
0.58	0	E2F7	CD44-AS1
0.58	0	CCL14	CARMN
0.58	0	ZCCHC12	CARMN
0.58	0	KRT3	CALML3-AS1
0.58	0	PLA2G4F	CALML3-AS1
0.58	0	SCN7A	CADM3-AS1
0.58	0	CHRDL1	ADAMTS9-AS2
0.58	0	MYOC	ADAMTS9-AS2
0.58	0	KLHL4	ACTA2-AS1
0.58	0	DSG1	AATBC
0.58	0	KRT74	AATBC
0.57	0	SLC34A1	SH3PXD2A-A
0.57	0	APOC2	PIK3CD-AS1
0.57	0	RNF225	OVOL1-AS1
0.57	0	CCL14	MRGPRF-AS1
0.57	0	TCF23	MRGPRF-AS1
0.57	0	APOC2	MMP2-AS1
0.57	0	DQX1	MIR9-3HG
0.57	0	DSG1	MIR4713HG
0.57	0	TMPRSS11F	MIR205HG
0.57	0	TCEAL2	MIR100HG
0.57	0	CASP12	MEF2C-AS1
0.57	0	CCL14	MAGI2-AS3
0.57	0	DPP6	MAGI2-AS3
0.57	0	GALNT17	MAGI2-AS3
0.57	0	GPIHBP1	MAGI2-AS3
0.57	0	KLHL4	MAGI2-AS3
0.57	0	KLC3	LINC02595
0.57	0	CRYBG2	LINC02541
0.57	0	APOC2	LINC02285
0.57	0	APOC2	LINC02099
0.57	0	FOXN1	LINC02031
0.57	0	TCF21	LINC01936
0.57	0	ZNF208	LINC01936
0.57	0	CCR8	LINC01684
0.57	0	TMPRSS11F	LINC01605
0.57	0	FOXN1	LINC01503
0.57	0	CCR8	LINC01094
0.57	0	CCR8	LINC00996
0.57	0	TMPRSS11F	LINC00964
0.57	0	TCF21	LINC00924
0.57	0	KRT3	LINC00640
0.57	0	E2F7	LINC00630

0.57	0	GAST	LINC00520
0.57	0	GSDMC	LINC00520
0.57	0	RNF225	IL20RB-AS1
0.57	0	ECRG4	HAND2-AS1
0.57	0	MYL3	EMX2OS
0.57	0	RGS22	EMX2OS
0.57	0	SLC34A1	DSG1-AS1
0.57	0	LCN6	CARMN
0.57	0	PRND	CARMN
0.57	0	SLC34A1	C10orf55
0.57	0	CASP12	ADAMTS9-AS2
0.57	0	LCN10	ADAMTS9-AS2
0.57	0	LCN6	ADAMTS9-AS2
0.57	0	WDR17	ADAMTS9-AS2
0.57	0	SCRG1	ADAMTS9-AS1
0.57	0	ZCCHC12	ADAMTS9-AS1
0.57	0	C7	ACTA2-AS1
0.57	0	TMPRSS11F	AATBC
0.56	0	OVOL1	SMILR
0.56	0	SLC34A1	SMILR
0.56	0	CASP12	RGS5
0.56	0	CRYBG2	PPP1R14B-A
0.56	0	KRT78	OVOL1-AS1
0.56	0	E2F7	OIP5-AS1
0.56	0	KLC3	MYOSLID
0.56	0	LRRC37A9P	MRPS31P5
0.56	0	SCN7A	MRGPRF-AS1
0.56	0	KRT3	MLIP-IT1
0.56	0	LCN6	MIR497HG
0.56	0	TMPRSS11F	MIR4713HG
0.56	0	AGTR1	MEG3
0.56	0	TCEAL2	MEG3
0.56	0	TCF21	MEG3
0.56	0	CRHBP	MEF2C-AS1
0.56	0	GPIHBP1	MEF2C-AS1
0.56	0	TCEAL2	MEF2C-AS1
0.56	0	RGS22	MAGI2-AS3
0.56	0	ZCCHC12	MAGI2-AS3
0.56	0	KRT16P1	LINC02595
0.56	0	GSDMC	LINC02560
0.56	0	DSG1	LINC02031
0.56	0	CCR8	LINC01934
0.56	0	SLC34A1	LINC01679
0.56	0	AGTR1	LINC01614

0.56	0	KRT31	LINC01605
0.56	0	CCR8	LINC01281
0.56	0	APOC2	LINC01094
0.56	0	GPIHBP1	LINC00924
0.56	0	CRYBG2	LINC00640
0.56	0	GSDMC	LINC00640
0.56	0	CRYBG2	LINC00520
0.56	0	KLC3	LINC00520
0.56	0	LCN10	HAND2-AS1
0.56	0	TCF23	HAND2-AS1
0.56	0	ZNF676	HAND2-AS1
0.56	0	FOXN1	GRHL3-AS1
0.56	0	DPP6	EMX2OS
0.56	0	OGN	EMX2OS
0.56	0	ZCCHC12	EMX2OS
0.56	0	TCEAL2	DNM3OS
0.56	0	E2F7	DLEU2L
0.56	0	TMPRSS11F	CT69
0.56	0	CRYBG2	CLCA3P
0.56	0	MAB21L3	CLCA3P
0.56	0	CRYBG2	CERS3-AS1
0.56	0	KLHL4	CARMN
0.56	0	TCF23	CARMN
0.56	0	CD300LG	CADM3-AS1
0.56	0	TMPRSS11F	C5rf34-AS1
0.56	0	GPIHBP1	ADAMTS9-AS2
0.56	0	GPR22	ADAMTS9-AS2
0.56	0	GALNT17	ADAMTS9-AS1
0.56	0	ZNF208	ADAMTS9-AS1
0.56	0	ZNF676	ADAMTS9-AS1
0.56	0	TCF23	ACTA2-AS1
0.56	0	E2F7	ACAP2-IT1
0.55	0	RGS22	ZNF781
0.55	0	LRRC37A9P	ZNF32-AS2
0.55	0	CCR8	TSPOAP1-AS
0.55	0	E2F7	TMPO-AS1
0.55	0	CKMT1B	SPINT1-AS1
0.55	0	LRRC37A9P	SMG7-AS1
0.55	0	RNF225	SH3PXD2A-A
0.55	0	OGN	RGS5
0.55	0	ZIC5	PCCA-DT
0.55	0	PLA2G4E	OVOL1-AS1
0.55	0	APOC2	OTOAP1
0.55	0	LRRC37A9P	NFYC-AS1

0.55	0	OVOL1	MLIP-IT1
0.55	0	CCL14	MIR497HG
0.55	0	CD300LG	MIR497HG
0.55	0	CRYBG2	MIR4713HG
0.55	0	PLA2G4E	MIR205HG
0.55	0	ZNF208	MEG3
0.55	0	CHRDL1	MEF2C-AS1
0.55	0	BPI	MAGI2-AS3
0.55	0	FOXN1	LINC02800
0.55	0	APOC2	LINC02611
0.55	0	GSDMC	LINC02595
0.55	0	KLC3	LINC02560
0.55	0	KRT74	LINC02541
0.55	0	AGTR1	LINC02202
0.55	0	OGN	LINC02202
0.55	0	KRT31	LINC02031
0.55	0	ZCCHC12	LINC01936
0.55	0	KRTAP4-1	LINC01633
0.55	0	GSDMC	LINC01605
0.55	0	UGT1A6	LINC01564
0.55	0	LGALS7B	LINC01527
0.55	0	GSDMC	LINC01503
0.55	0	MYL3	LINC00924
0.55	0	PRND	LINC00924
0.55	0	TCF21	LINC00578
0.55	0	FAM83B	LINC00520
0.55	0	DSG1	LINC00519
0.55	0	FOXE1	LINC00519
0.55	0	E2F7	LINC00511
0.55	0	FOXE1	KRT42P
0.55	0	FOXE1	KRT16P3
0.55	0	LGALS7B	KRT16P3
0.55	0	KRT78	IL20RB-AS1
0.55	0	CCL14	HID1-AS1
0.55	0	SCRG1	HAND2-AS1
0.55	0	E2F7	GRHL3-AS1
0.55	0	MCIDAS	GOLGA2P5
0.55	0	GSDMC	GBP1P1
0.55	0	FOXN1	FMO9P
0.55	0	E2F7	EBLN3P
0.55	0	GALNT17	DNM3OS
0.55	0	KRT78	CLCA3P
0.55	0	APOC2	CEP250-AS1
0.55	0	CASQ2	CARMN

0.55	0	DPP6	CARMN
0.55	0	GALNT17	CARMN
0.55	0	MEOX2	CARMN
0.55	0	SCRG1	CARMN
0.55	0	ZNF676	CARMN
0.55	0	LGALS7B	CALML3-AS1
0.55	0	CCL16	CADM3-AS1
0.55	0	MEOX2	CADM3-AS1
0.55	0	FAM83B	C10orf55
0.55	0	FAM83B	ATP1B3-AS1
0.55	0	LRRC37A9P	ANKRD10-IT
0.55	0	DPP6	ADAMTS9-AS2
0.55	0	ECRG4	ADAMTS9-AS2
0.55	0	KLHL33	ADAMTS9-AS2
0.55	0	CASP12	ACTA2-AS1
0.55	0	CHRDL1	ACTA2-AS1
0.55	0	PRND	ACTA2-AS1
0.55	0	TCEAL2	A2M-AS1
0.54	0	OGN	ZNF781
0.54	0	CCR8	TRG-AS1
0.54	0	FAM83B	SMILR
0.54	0	FOXN1	SMILR
0.54	0	KRT31	SMILR
0.54	0	SLC34A1	SMAD5-AS1
0.54	0	FAM83B	SH3PXD2A-A
0.54	0	MCIDAS	RAET1K
0.54	0	TCEAL2	PTPRD-AS1
0.54	0	FAM83B	PLA2G4E-AS
0.54	0	C7	MRGPRF-AS1
0.54	0	CASP12	MRGPRF-AS1
0.54	0	FAM83B	MLIP-IT1
0.54	0	KRT31	MLIP-IT1
0.54	0	PLA2G4E	MLIP-IT1
0.54	0	TCEAL2	MIR497HG
0.54	0	TCF23	MIR497HG
0.54	0	KRT16P1	MIR3659HG
0.54	0	DSG1	MIR205HG
0.54	0	OGN	MIR100HG
0.54	0	MYL3	MEG9
0.54	0	ZCCHC12	MEF2C-AS1
0.54	0	ECRG4	MAGI2-AS3
0.54	0	ZNF676	MAGI2-AS3
0.54	0	FOXN1	LINC02595
0.54	0	LGALS7B	LINC02560

0.54	0	OVOL1	LINC02560
0.54	0	TMPRSS11F	LINC02560
0.54	0	DSG1	LINC02541
0.54	0	GSDMC	LINC02178
0.54	0	APOC2	LINC02073
0.54	0	CCR8	LINC01943
0.54	0	CASQ2	LINC01936
0.54	0	CCR8	LINC01857
0.54	0	FOXN1	LINC01752
0.54	0	E2F7	LINC01572
0.54	0	KRT78	LINC01269
0.54	0	CCR8	LINC01215
0.54	0	DSG1	LINC00964
0.54	0	EPGN	LINC00941
0.54	0	CRYBG2	LINC00887
0.54	0	KRT78	LINC00640
0.54	0	OVOL1	LINC00520
0.54	0	KRT16P1	LINC00519
0.54	0	KRT16P1	KRT42P
0.54	0	OVOL1	KRT16P3
0.54	0	CCR8	IL21-AS1
0.54	0	KLC3	HOXC13-AS
0.54	0	HFM1	HHIP-AS1
0.54	0	CCR8	HECW2-AS1
0.54	0	AGTR1	HAND2-AS1
0.54	0	GALNT17	HAND2-AS1
0.54	0	CRYBG2	GRHL3-AS1
0.54	0	SLC34A1	GRHL3-AS1
0.54	0	FAM83B	EGFR-AS1
0.54	0	KRT74	DSG1-AS1
0.54	0	PLA2G4E	DSG1-AS1
0.54	0	OVOL1	CLCA3P
0.54	0	TCF21	CCDC144NL-
0.54	0	LCN10	CARMN
0.54	0	FAM83B	CALML3-AS1
0.54	0	ADH1B	CADM3-AS1
0.54	0	RNF225	ATP1B3-AS1
0.54	0	APOC2	ADPGK-AS1
0.54	0	CCR8	ADPGK-AS1
0.54	0	CRHBP	ADAMTS9-AS2
0.54	0	RGS22	ADAMTS9-AS2
0.54	0	SYT9	ADAMTS9-AS2
0.54	0	ZCCHC12	ADAMTS9-AS2
0.54	0	ZNF208	ACTA2-AS1

0.53	0	GPIHBP1	ZNF781
0.53	0	MYL3	ZNF781
0.53	0	E2F7	UBE2Q2P2
0.53	0	APOC2	TRG-AS1
0.53	0	E2F7	TNFRSF10A-
0.53	0	CKMT1B	STRCP1
0.53	0	FOXN1	SPATA41
0.53	0	KRT3	SMILR
0.53	0	KRT16P1	SH3PXD2A-A
0.53	0	LRRC37A9P	SAP30L-AS1
0.53	0	AGTR1	RGS5
0.53	0	CCL14	RGS5
0.53	0	E2F7	RAET1K
0.53	0	RNF225	PLA2G4E-AS
0.53	0	LRRC37A9P	NPTN-IT1
0.53	0	APOC2	NCF1B
0.53	0	MYOZ2	MRGPRF-AS1
0.53	0	KRT16P1	MLIP-IT1
0.53	0	ZCCHC12	MIR497HG
0.53	0	DSG1	MIR3659HG
0.53	0	FOXE1	MIR205HG
0.53	0	KRT3	MIR205HG
0.53	0	KRT31	MIR205HG
0.53	0	APOC2	MIR155HG
0.53	0	AGTR1	MIR100HG
0.53	0	MYL3	MIR100HG
0.53	0	MEOX2	MEF2C-AS1
0.53	0	LCN6	MAGI2-AS3
0.53	0	MYOZ2	MAGI2-AS3
0.53	0	PRND	MAGI2-AS3
0.53	0	KLRG2	LINC02800
0.53	0	KRT78	LINC02595
0.53	0	SLC34A1	LINC02595
0.53	0	DQX1	LINC02541
0.53	0	KLC3	LINC02541
0.53	0	KRT16P1	LINC02541
0.53	0	C7	LINC02202
0.53	0	CRYBG2	LINC02031
0.53	0	BPI	LINC01936
0.53	0	CHRDL1	LINC01936
0.53	0	OGN	LINC01936
0.53	0	KRT3	LINC01605
0.53	0	SLC34A1	LINC01605
0.53	0	RNF225	LINC01527

0.53	0	SLC34A1	LINC01527
0.53	0	C7	LINC00924
0.53	0	ZNF676	LINC00924
0.53	0	E2F7	LINC00265
0.53	0	FOXN1	KRT42P
0.53	0	FOXN1	HOXC13-AS
0.53	0	CD300LG	HAND2-AS1
0.53	0	MYOZ2	HAND2-AS1
0.53	0	SYT9	HAND2-AS1
0.53	0	DQX1	GRHL3-AS1
0.53	0	OVOL1	GRHL3-AS1
0.53	0	CRYBG2	FMO9P
0.53	0	APOC2	EML4-AS1
0.53	0	LRRC37A9P	EDRF1-AS1
0.53	0	FAM83B	DSG1-AS1
0.53	0	GSDMC	DSG1-AS1
0.53	0	PLA2G4F	DSG1-AS1
0.53	0	E2F7	DLEU2
0.53	0	LGALS7B	CERS3-AS1
0.53	0	DQX1	CDKN2B-AS1
0.53	0	GPR22	CARMN
0.53	0	PLA2G4E	CALML3-AS1
0.53	0	CHRDL1	CADM3-AS1
0.53	0	SLC34A1	C5rf34-AS1
0.53	0	FAM83B	C2CD4D-AS1
0.53	0	PADI3	C10orf55
0.53	0	LRRC37A9P	AP4B1-AS1
0.53	0	LRRC37A9P	ALG13-AS1
0.53	0	CCL16	ADAMTS9-AS2
0.53	0	OPCML	ADAMTS9-AS2
0.53	0	ZNF99	ADAMTS9-AS2
0.53	0	CASP12	ADAMTS9-AS1
0.53	0	CASQ2	ADAMTS9-AS1
0.53	0	LCN10	ADAMTS9-AS1
0.53	0	PRND	ADAMTS9-AS1
0.53	0	RGS22	ADAMTS9-AS1
0.53	0	ZCCHC12	ACTA2-AS1
0.53	0	CHRDL1	A2M-AS1
0.53	0	HFM1	A2M-AS1
0.53	0	ZCCHC12	A2M-AS1
0.52	0	C7	ZNF781
0.52	0	PRND	ZNF781
0.52	0	ZCCHC12	ZNF781
0.52	0	RGS22	WT1-AS

0.52	0	LRRC37A9P	STAG3L5P
0.52	0	KRT16P1	SMILR
0.52	0	MAB21L3	SMILR
0.52	0	PADI3	SMILR
0.52	0	GAST	SH3PXD2A-A
0.52	0	MYL3	RGS5
0.52	0	ZCCHC12	RGS5
0.52	0	CCR8	PCED1B-AS1
0.52	0	E2F7	OGFRP1
0.52	0	LRRC37A9P	NDUFA6-DT
0.52	0	CCR8	NCF1B
0.52	0	LRRC37A9P	NARF-IT1
0.52	0	CHRDL1	MRGPRF-AS1
0.52	0	GPIHBP1	MRGPRF-AS1
0.52	0	SCRG1	MRGPRF-AS1
0.52	0	ZNF208	MRGPRF-AS1
0.52	0	GSDMC	MIR3659HG
0.52	0	GPM6A	MEF2C-AS1
0.52	0	KLHL33	MAGI2-AS3
0.52	0	SCRG1	MAGI2-AS3
0.52	0	KRT74	LINC02800
0.52	0	SLC34A1	LINC02784
0.52	0	DSG1	LINC02595
0.52	0	FAM83B	LINC02595
0.52	0	FOXE1	LINC02595
0.52	0	KRT74	LINC02595
0.52	0	KRT16P1	LINC02560
0.52	0	KRT3	LINC02560
0.52	0	CCR8	LINC02528
0.52	0	CCR8	LINC02397
0.52	0	KLHL4	LINC02202
0.52	0	KRT16P1	LINC02137
0.52	0	SLC34A1	LINC02137
0.52	0	AGTR1	LINC01936
0.52	0	GALNT17	LINC01936
0.52	0	RGS22	LINC01936
0.52	0	SLC34A1	LINC01752
0.52	0	NEIL3	LINC01572
0.52	0	GPIHBP1	LINC01352
0.52	0	APOC2	LINC01281
0.52	0	DSG1	LINC01010
0.52	0	CRYBG2	LINC00964
0.52	0	GSDMC	LINC00964
0.52	0	KRT16P1	LINC00964

0.52	0	ZNF208	LINC00924
0.52	0	KRT3	LINC00887
0.52	0	KLHL4	LINC00702
0.52	0	UGT1A6	LINC00519
0.52	0	CRYBG2	LINC00511
0.52	0	DQX1	LINC00511
0.52	0	GAST	KRT42P
0.52	0	GSDMC	KRT42P
0.52	0	KRT3	KRT16P3
0.52	0	KRT31	KRT16P3
0.52	0	GSDMC	HOXC13-AS
0.52	0	GPIHBP1	HID1-AS1
0.52	0	LRRC37A9P	HEXD-IT1
0.52	0	CRHBP	HAND2-AS1
0.52	0	GPM6A	HAND2-AS1
0.52	0	TCF21	HAND2-AS1
0.52	0	E2F7	FMR1-IT1
0.52	0	PLA2G4E	FAM83A-AS1
0.52	0	ZNF208	FAM218A
0.52	0	ECRG4	EMX2OS
0.52	0	ZNF208	EMX2OS
0.52	0	RCVRN	DNM3OS
0.52	0	ZNF208	DNM3OS
0.52	0	LRRC37A9P	CTBP1-DT
0.52	0	PLA2G4E	CT69
0.52	0	DSG1	CLCA3P
0.52	0	FOXE1	CERS3-AS1
0.52	0	KLRG2	CERS3-AS1
0.52	0	PLA2G4E	CERS3-AS1
0.52	0	PLA2G4F	CERS3-AS1
0.52	0	OPCML	CARMN
0.52	0	WDR17	CARMN
0.52	0	KLC3	CALML3-AS1
0.52	0	KLRG2	CALML3-AS1
0.52	0	GPR22	CADM3-AS1
0.52	0	DSG1	C5rf34-AS1
0.52	0	KRT74	C5rf34-AS1
0.52	0	FOXN1	C10orf55
0.52	0	E2F7	ATP1B3-AS1
0.52	0	C1QTNF9	ADAMTS9-AS2
0.52	0	MYL3	ADAMTS9-AS2
0.52	0	TCF23	ADAMTS9-AS2
0.52	0	DPP6	ACTA2-AS1
0.52	0	GALNT17	ACTA2-AS1

0.52	0	LGALS7B	AATBC
0.52	0	PLA2G4F	AATBC
0.52	0	SYT9	A2M-AS1
0.51	0	LRRC37A9P	XIST
0.51	0	CHRDL1	WT1-AS
0.51	0	ECRG4	WT1-AS
0.51	0	MYL3	WT1-AS
0.51	0	TCF23	WT1-AS
0.51	0	ZCCHC12	WT1-AS
0.51	0	NMU	UNC5B-AS1
0.51	0	LRRC37A9P	UGDH-AS1
0.51	0	LRRC37A9P	STARD4-AS1
0.51	0	C5rf66-AS1	SOX21-AS1
0.51	0	RNF225	SMILR
0.51	0	KLC3	SH3PXD2A-A
0.51	0	KRT3	SH3PXD2A-A
0.51	0	KRT31	SH3PXD2A-A
0.51	0	AGTR1	PTPRD-AS1
0.51	0	OGN	PTPRD-AS1
0.51	0	SCN7A	PTPRD-AS1
0.51	0	ZCCHC12	PTPRD-AS1
0.51	0	OVOL1	PLA2G4E-AS
0.51	0	KRT16P1	OVOL1-AS1
0.51	0	MAB21L3	OVOL1-AS1
0.51	0	LCN10	MRGPRF-AS1
0.51	0	KRT74	MLIP-IT1
0.51	0	C7	MIR497HG
0.51	0	MYL3	MIR497HG
0.51	0	GSDMC	MIR4713HG
0.51	0	KRT3	MIR4713HG
0.51	0	KRT74	MIR4713HG
0.51	0	KRTAP4-1	MIR4713HG
0.51	0	OVOL1	MIR4713HG
0.51	0	SLC34A1	MIR4713HG
0.51	0	KRT74	MIR3659HG
0.51	0	CRYBG2	MIR222HG
0.51	0	KRT16P1	MIR222HG
0.51	0	KLHL4	MIR100HG
0.51	0	GPIHBP1	MEG3
0.51	0	OGN	MEG3
0.51	0	BPI	MEF2C-AS1
0.51	0	LCN6	MEF2C-AS1
0.51	0	PRND	MEF2C-AS1
0.51	0	SCN7A	MEF2C-AS1

0.51	0	SCRG1	MEF2C-AS1
0.51	0	NPAS4	MBNL1-AS1
0.51	0	CRHBP	MAGI2-AS3
0.51	0	RCVRN	MAGI2-AS3
0.51	0	WDR17	MAGI2-AS3
0.51	0	APOC2	MACORIS
0.51	0	KRT31	LINC02595
0.51	0	FOXE1	LINC02560
0.51	0	PLA2G4E	LINC02560
0.51	0	LGALS7B	LINC02541
0.51	0	APOC2	LINC02391
0.51	0	MYL3	LINC02202
0.51	0	TCF21	LINC02202
0.51	0	KLC3	LINC02178
0.51	0	DSG1	LINC02137
0.51	0	GPIHBP1	LINC01936
0.51	0	TCF23	LINC01936
0.51	0	APOC2	LINC01934
0.51	0	KRT3	LINC01752
0.51	0	CRYBG2	LINC01679
0.51	0	KLHL4	LINC01614
0.51	0	FOXN1	LINC01605
0.51	0	FAM83B	LINC01527
0.51	0	LRRC37A9P	LINC01521
0.51	0	DSG1	LINC01395
0.51	0	APOC2	LINC01150
0.51	0	HFM1	LINC01122
0.51	0	CASP12	LINC00987
0.51	0	APOC2	LINC00892
0.51	0	TCF21	LINC00702
0.51	0	PLA2G4F	LINC00640
0.51	0	LRRC37A9P	LINC00624
0.51	0	APOC2	LINC00426
0.51	0	CCR8	LINC00158
0.51	0	APOC2	LCT-AS1
0.51	0	LRRC37A9P	KIAA1671-A
0.51	0	KRT3	IL20RB-AS1
0.51	0	KLC3	IGFL2-AS1
0.51	0	OGN	HID1-AS1
0.51	0	PRND	HAND2-AS1
0.51	0	LRRC37A9P	GBAP1
0.51	0	GSDMC	FMO9P
0.51	0	SLC34A1	FMO9P
0.51	0	MYOC	EMX2OS

0.51	0	LRRC37A9P	EBLN3P
0.51	0	CALML5	DSG1-AS1
0.51	0	MAB21L3	DSG1-AS1
0.51	0	KIAA1210	DNM3OS
0.51	0	KRT16P1	CT69
0.51	0	FAM83B	CLCA3P
0.51	0	KRT31	CLCA3P
0.51	0	KRT3	CERS3-AS1
0.51	0	E2F7	CDKN2B-AS1
0.51	0	CRYBG2	CDKN2A-DT
0.51	0	GSDMC	CDKN2A-DT
0.51	0	CCR8	CCR5AS
0.51	0	CRHBP	CARMN
0.51	0	ECRG4	CARMN
0.51	0	MYOZ2	CARMN
0.51	0	KRTAP4-1	CALML3-AS1
0.51	0	BPI	CADM3-AS1
0.51	0	CRHBP	CADM3-AS1
0.51	0	FAM83B	C5rf34-AS1
0.51	0	FOXE1	C5rf34-AS1
0.51	0	KLRG2	C5rf34-AS1
0.51	0	LRRC37A9P	BMS1P1
0.51	0	BPI	ADAMTS9-AS1
0.51	0	GPM6A	ADAMTS9-AS1
0.51	0	GPR22	ADAMTS9-AS1
0.51	0	OPCML	ADAMTS9-AS1
0.51	0	LCN6	ACTA2-AS1
0.51	0	OPCML	ACTA2-AS1
0.5	0	BPI	ZNF781
0.5	0	CCL14	ZNF781
0.5	0	TCEAL2	ZNF781
0.5	0	ZNF208	ZNF781
0.5	0	ZNF676	ZNF781
0.5	0	ECRG4	ZNF667-AS1
0.5	0	ZNF208	ZNF300P1
0.5	0	BPI	WT1-AS
0.5	0	OGN	WT1-AS
0.5	0	APOC2	USP30-AS1
0.5	0	FAM83B	TNFRSF10A-
0.5	0	FOXN1	STEAP3-AS1
0.5	0	LGALS7B	STEAP3-AS1
0.5	0	FOXE1	SPATA41
0.5	0	LRRC37A9P	SPAG5-AS1
0.5	0	CCR8	SOCAR

0.5	0	DQX1	SH3PXD2A-A
0.5	0	KRTAP4-1	SH3PXD2A-A
0.5	0	MAB21L3	SH3PXD2A-A
0.5	0	GSDMC	OVOL1-AS1
0.5	0	LRRC37A9P	OBI1-AS1
0.5	0	SEL1L2	MRGPRF-AS1
0.5	0	ZCCHC12	MRGPRF-AS1
0.5	0	CASP12	MIR497HG
0.5	0	LRRC37A9P	MIR4453HG
0.5	0	KRT74	MIR222HG
0.5	0	TMPRSS11F	MIR222HG
0.5	0	KRT78	MIR205HG
0.5	0	CCR8	MIR155HG
0.5	0	DPP6	MIR100HG
0.5	0	E2F7	MFSD14C
0.5	0	RCVRN	MEG3
0.5	0	LCN10	MEF2C-AS1
0.5	0	E2F7	MCM3AP-AS1
0.5	0	OGN	MBNL1-AS1
0.5	0	LCN10	MAGI2-AS3
0.5	0	SLC34A1	LINC02800
0.5	0	APOC2	LINC02694
0.5	0	KRT3	LINC02595
0.5	0	DQX1	LINC02560
0.5	0	DSG1	LINC02560
0.5	0	KLRG2	LINC02541
0.5	0	KRT31	LINC02541
0.5	0	CCL14	LINC02202
0.5	0	CHRDL1	LINC02202
0.5	0	LCN10	LINC02202
0.5	0	LCN6	LINC02202
0.5	0	SCN7A	LINC02202
0.5	0	KRT78	LINC02178
0.5	0	PLA2G4E	LINC02178
0.5	0	KRT74	LINC02031
0.5	0	PADI3	LINC02031
0.5	0	SCN7A	LINC01936
0.5	0	LRRC37A9P	LINC01719
0.5	0	KRT16P1	LINC01679
0.5	0	FAM83B	LINC01605
0.5	0	KRT78	LINC01605
0.5	0	GAST	LINC01527
0.5	0	KLC3	LINC01527
0.5	0	MYL3	LINC01352

0.5	0	PLA2G4E	LINC01010
0.5	0	CRHBP	LINC00987
0.5	0	KLRG2	LINC00964
0.5	0	KRT78	LINC00964
0.5	0	LRRC37A9P	LINC00954
0.5	0	CCL14	LINC00924
0.5	0	KRT78	LINC00887
0.5	0	KLRG2	LINC00885
0.5	0	AGTR1	LINC00702
0.5	0	FAM83B	LINC00640
0.5	0	FOXE1	LINC00640
0.5	0	PADI3	LINC00640
0.5	0	RGS22	LINC00639
0.5	0	AGTR1	LINC00578
0.5	0	KRT78	LINC00520
0.5	0	MYL3	LEF1-AS1
0.5	0	CRYBG2	KRT42P
0.5	0	KLC3	KRT42P
0.5	0	C5rf66-AS1	KRT16P3
0.5	0	FAM83B	KRT16P3
0.5	0	KLC3	KRT16P3
0.5	0	DQX1	IL20RB-AS1
0.5	0	KRT74	IL20RB-AS1
0.5	0	KRT3	IGFL2-AS1
0.5	0	DSG1	GRHL3-AS1
0.5	0	KRT74	GRHL3-AS1
0.5	0	E2F7	GHET1
0.5	0	TMPRSS11F	FMO9P
0.5	0	FAM83B	FAM83A-AS1
0.5	0	RNF225	FAM157C
0.5	0	LRRC37A9P	EP300-AS1
0.5	0	GPIHBP1	DNM3OS
0.5	0	KLHL4	DNM3OS
0.5	0	PRND	DNM3OS
0.5	0	MCIDAS	DDX11-AS1
0.5	0	LRRC37A9P	CTBP1-AS
0.5	0	FOXN1	CLCA3P
0.5	0	KRT31	CERS3-AS1
0.5	0	APOC2	CCR5AS
0.5	0	ADH1B	CARMN
0.5	0	BPI	CARMN
0.5	0	SEL1L2	CARMN
0.5	0	PADI3	CALML3-AS1
0.5	0	RNF225	CALML3-AS1

0.5	0	CASP12	CADM3-AS1
0.5	0	CMA1	CADM3-AS1
0.5	0	RGS22	CADM3-AS1
0.5	0	GSDMC	C2CD4D-AS1
0.5	0	FOXE1	C10orf55
0.5	0	OVOL1	C10orf55
0.5	0	TMPRSS11F	C10orf55
0.5	0	AGTR1	ADAMTS9-AS2
0.5	0	GALNT17	ADAMTS9-AS2
0.5	0	MAS1	ADAMTS9-AS2
0.5	0	MEOX2	ADAMTS9-AS2
0.5	0	ADH1B	ADAMTS9-AS1
0.5	0	TCF21	ADAMTS9-AS1
0.5	0	CASQ2	ACTA2-AS1
0.5	0	CRHBP	ACTA2-AS1
0.5	0	MYOZ2	ACTA2-AS1
0.5	0	KRT16P1	AATBC
0.5	0	KRT31	AATBC

TableS7.33-lncRNA-CESC random forest model coefficients

symbol	x
LINC02783	0.03684915
EGFR-AS1	0.03595983
SPRY4-AS1	0.03434902
DAAM2-AS1	0.03007755
APCDD1L-DT	0.029287
ITGB1-DT	0.02655513
LINC01929	0.02153229
HS1BP3-IT1	0.0197866
LINC02015	0.01884213
LINC02802	0.01725846
BASP1-AS1	0.01276879
STXBP5-AS1	0.01207628
DDN-AS1	0.01200521
NADK2-AS1	0.01118599
LINC00460	0.01065605
STARD4-AS1	0.00991287
LINC02818	0.00898744
SH3RF3-AS1	0.00835686
LNCOG	0.00830693
HIF1A-AS3	0.00788231
LINC01615	0.00747224
PTPRD-AS1	0.00645904
FLNB-AS1	0.00631845

INHBA-AS1	0.00477924
LINC01213	0.00341016
HCG15	0.0012277
UBE2Q2P1	0.00068536
LINC02551	-9.75E-05
LINC02544	-0.0005267
LINC01719	-0.00096
LINC00882	-0.0011737
LINC01235	-0.0013615
WWC2-AS2	-0.0018596

TableS8.Multivariate Cox regression coefficient

	coef	exp(coef)	se(coef)	z	Pr(>|z|)	
Score	1.87E-01	1.21E+00	2.53E-02	7.384	1.54E-13	***
clinical_stagestage II	-2.60E-01	7.71E-01	4.77E-01	-0.546	0.58538	
clinical_stagestage III	6.02E-02	1.06E+00	4.50E-01	0.134	0.89348	
clinical_stagestage IV	4.37E-01	1.55E+00	5.04E-01	0.868	0.38529	
clinical_stagestage NA	-1.52E+01	2.65E-07	3.01E+03	-0.005	0.99599	
neoplasm_histologic_gradeG2	-4.32E-02	9.58E-01	6.67E-01	-0.065	0.94837	
neoplasm_histologic_gradeG3	-4.41E-01	6.44E-01	6.86E-01	-0.643	0.52048	
neoplasm_histologic_gradeG4	-1.57E+01	1.57E-07	4.33E+03	-0.004	0.99711	
neoplasm_histologic_gradeGX	-5.45E-01	5.80E-01	7.82E-01	-0.697	0.48593	
pathologic_MM1	2.04E-01	1.23E+00	6.87E-01	0.297	0.76664	
pathologic_MMX	5.94E-01	1.81E+00	3.45E-01	1.724	0.08471	.
pathologic_TT2	-2.23E-01	8.00E-01	5.01E-01	-0.444	0.65687	
pathologic_TT3	8.87E-01	2.43E+00	5.85E-01	1.516	0.12947	
pathologic_TT4	1.07E+00	2.90E+00	7.12E-01	1.497	0.13445	
pathologic_TTX	-4.14E-01	6.61E-01	5.03E-01	-0.822	0.41097	
pathologic_TTis	-1.66E+01	6.31E-08	1.19E+04	-0.001	0.99889	
pathologic_NN1	9.99E-01	2.72E+00	3.78E-01	2.641	0.00825	**
pathologic_NNX	6.46E-01	1.91E+00	5.21E-01	1.241	0.21453	
